# Collagen VI expression is negatively mechanosensitive in pancreatic cancer cells and supports the metastatic niche

**DOI:** 10.1242/jcs.259978

**Published:** 2022-12-22

**Authors:** Vasileios Papalazarou, James Drew, Amelie Juin, Heather J. Spence, Jamie Whitelaw, Colin Nixon, Manuel Salmeron-Sanchez, Laura M. Machesky

**Affiliations:** ^1^ Cancer Research UK Beatson Institute, Switchback Road, Bearsden, Glasgow G61 1BD, UK; ^2^ Centre for the Cellular Microenvironment, University of Glasgow, Glasgow G11 6EW, UK; ^3^ Institute of Cancer Sciences, University of Glasgow, Glasgow G61 1BD, UK

**Keywords:** Extracellular matrix, Mechanosensing, Cancer metastasis, Collagen VI, Pancreatic cancer, Integrin adhesion

## Abstract

Pancreatic cancer is a deadly and highly metastatic disease, although how metastatic lesions establish is not fully understood. A key feature of pancreatic tumours is extensive fibrosis and deposition of extracellular matrix (ECM). While pancreatic cancer cells are programmed by stimuli derived from a stiff ECM, metastasis requires loss of attachment and adaptation to a softer microenvironment at distant sites. Growing evidence suggests that stiff ECM influences pancreatic cancer cell behaviour. Here, we argue that this influence is reversible and that pancreatic cancer cells can be reprogrammed upon sensing soft substrates. Using engineered polyacrylamide hydrogels with tuneable mechanical properties, we show that collagen VI is specifically upregulated in pancreatic cancer cells on soft substrates, due to a lack of integrin engagement. Furthermore, the expression of collagen VI is inversely correlated with mechanosensing and activity of YAP (also known as YAP1), which might be due to a direct or indirect effect on transcription of genes encoding collagen VI. Collagen VI supports migration *in vitro* and metastasis formation *in vivo*. Metastatic nodules formed by pancreatic cancer cells lacking *Col6a1* display stromal cell-derived collagen VI deposition, suggesting that collagen VI derived from either cancer cells or the stroma is an essential component of the metastatic niche.

This article has an associated First Person interview with Vasileios Papalazarou, joint first author of the paper.

## Teaser

**Summary:** Collagen VI is expressed by pancreatic tumours and metastases in a mechanosensitive way to promote niche colonisation.

## INTRODUCTION

The dissemination of malignant cells from primary tumours to distant sites and the formation of metastatic nodules is a key step in cancer progression and aggressiveness. An essential component of the metastatic niche is the extracellular matrix (ECM) – the collection of extracellular proteins that provide the three-dimensional (3D) scaffold within which cells organise to form complex structures. It is emerging that cancer cell–ECM interactions have important roles in the establishment of metastatic tumours at all stages of the metastatic cascade (Drew and Machesky, 2021). These interactions depend on not only the biological components of the ECM but also its physical and mechanical properties.

The ability of cancer cells to respond to the physical properties of their surroundings is increasingly recognised as crucial to disease progression (Broders-Bondon et al., 2018). In solid tumours such as pancreatic ductal adenocarcinoma (PDAC), deposition of ECM leads to extensive matricellular fibrosis that is linked to cancer aggressiveness (Mahadevan and Von Hoff, 2007; Perez et al., 2021) and contributes to its dismal 5-year survival rates (Siegel et al., 2021). Increased tissue stiffness has been shown to drive extravasation from the primary tumour through initiating epithelial–mesenchymal transition (EMT) in PDAC cells and stimulating invasion (Rice et al., 2017). However, cells escaping the primary tumour must survive and grow in low-adhesion and soft tissue environments, such as the liver and lung, to form metastatic lesions (Yachida and Iacobuzio-Donahue, 2009). Given that metastatic dissemination is the leading cause of death in pancreatic cancer (Balaban et al., 2017), understanding the adaptations that PDAC cells undergo during these transitions is of significant importance. Specifically, whether sensing of stiff ECM irreversibly modulates cancer cell behaviour or whether cancer cells can actively respond to low-stiffness stimuli remains unclear.

Increased tissue stiffness and tension affects cancer cell identity and behaviour through complex signalling pathways that are collectively referred to as ‘mechanosensation’ (Papalazarou et al., 2018). The fundamental process of mechanosensation involves integrin receptor engagement of extracellular substrates, actin cytoskeleton remodelling and activation of key transcriptional regulators such as Yes-associated protein 1 (referred to hereafter as YAP, encoded by *Yap1*) (Panciera et al., 2017). These mechanosensitive pathways influence the directional migration (Laklai et al., 2016), metabolism (Papalazarou et al., 2020) and chemoresistance (Rice et al., 2017) of PDAC cells. Although some fundamental components of these pathways have been elucidated, much is still unknown about how mechanosensitive pathways are utilised by pancreatic cancer cells at different stages of disease progression.

In primary PDAC, the majority of the ECM is deposited by stromal cells [in particular cancer-associated fibroblasts (CAFs)] that secrete large amounts of fibrillar ECM components such as fibronectin and collagen I, increasing tissue stiffness (Elyada et al., 2019). Unfortunately, approaches aimed at targeting ECM deposition in mouse models of PDAC (Özdemir et al., 2014) and clinical trials (Doherty et al., 2018) have proven unsuccessful, likely due to the loss of the ECM as a physical barrier to restrain the tumour (Bhattacharjee et al., 2021; Jiang et al., 2020). Therefore, a more nuanced understanding of the relationship between ECM deposition, tissue stiffness and PDAC development is required.

Recent proteomic studies have revealed that alongside CAFs, cancer cells themselves produce a wide range of ECM proteins that contribute to the tumour microenvironment (Tian et al., 2019). Indeed, expression of cancer cell-derived ECM proteins correlates with poor survival in PDAC (Tian et al., 2019), suggesting that these proteins exert important roles in tumour progression. These functions may have relevance in metastatic dissemination, where cancer cells must quickly form a microenvironment to support their survival and growth (Drew and Machesky, 2021; Jiang et al., 2020). Indeed, a recent study has shown that various PDAC-derived ECM components have roles in supporting metastatic dissemination (Tian et al., 2020); although our understanding of the functional roles of PDAC-derived ECM, and the mechanisms controlling its expression, remains limited. Given the intimate relationship between ECM and tissue stiffness, it is also unclear whether PDAC cells regulate their own ECM production in response to changes in their physical environment.

In this study, we define a link between substrate stiffness, PDAC ECM expression and metastatic potential. By combining the use of mechanically tuneable hydrogels with RNA sequencing transcriptomic analyses, we observed that in environments of reduced stiffness pancreatic cancer cells upregulate the expression of genes involved in ECM production. This was prominently manifested by upregulation of three genes involved in collagen VI synthesis: *Col6a1*, *Col6a2* and *Col6a3*. Focusing on collagen VI, we show that collagen VI upregulation is a response to a lack of integrin-based mechanosensation and diminished YAP nuclear localisation on soft substrates. Furthermore, we find that PDAC cell-derived collagen VI supports invasive behaviour *in vitro* and metastatic potential of pancreatic cancer cells *in vivo*.

## RESULTS

### Matrix stiffness alters pancreatic cancer cell expression of ECM proteins

**Fig. 1.**
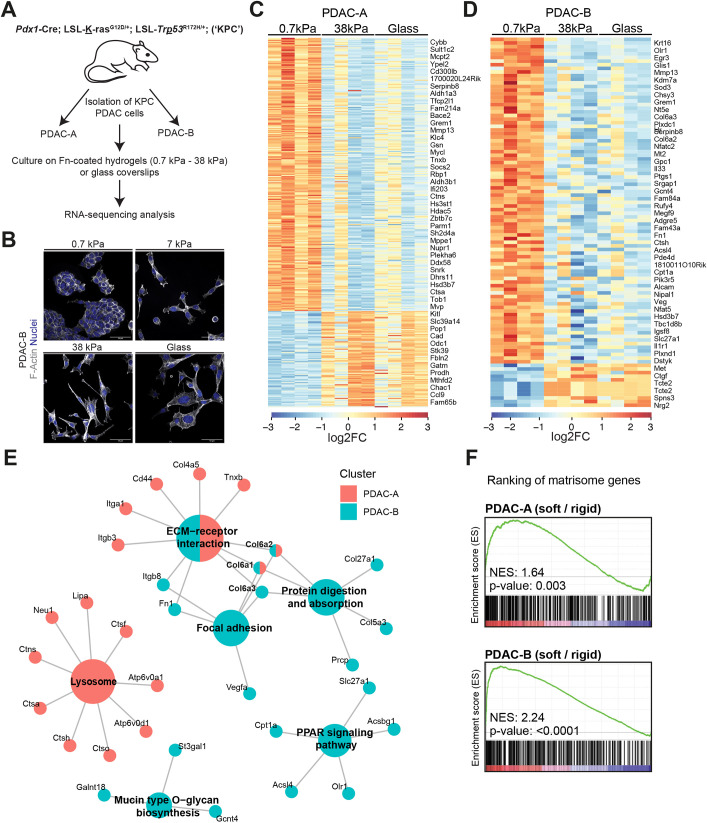
**Low substrate stiffness promotes the expression of matrisome-related genes in PDAC cells.** (A) Schematic representation of the RNA sequencing strategy for analysis of PDAC cells. PDAC-A and PDAC-B cell lines isolated from the *Pdx1*-Cre; LSL-*Kras*^G12D/+^; LSL-*Trp53*^R172H/+^ (KPC) mouse model were cultured on 0.7 kPa, 7 kPa or 38 kPa fibronectin (Fn)-coated polyacrylamide hydrogels or Fn-coated glass coverslips for 24 h. Total RNA was extracted, and RNA sequencing was performed. (B) Immunofluorescence of PDAC-B cells cultured on 0.7 kPa, 7 kPa or 38 kPa Fn-coated polyacrylamide hydrogels or Fn-coated glass coverslips, as indicated, showing F-actin (phalloidin, grey) and nuclei (DAPI, blue). Images are representative of three independent experiments. Scale bars: 50 μm. (C,D) Expression heatmaps of differentially expressed genes in cells cultured on 0.7 kPa and 38 kPa hydrogels, and on glass coverslips, as described in A. In PDAC-A cells (C), 258 genes were upregulated and 92 downregulated in the 0.7 kPa samples. In PDAC-B cells (D), 90 genes were upregulated and 10 downregulated in the 0.7 kPa samples (*P*_adj_<0.05; log_2_ fold change >1). In both cell lines, cells cultured on glass coverslips show similarity in expression pattern with cells cultured on 38 kPa hydrogels. Data is from *n*=4 independent replicates per condition for each cell line, with each replicate shown as a column in the heatmap. FC, fold change. (E) CNET plot of KEGG pathway analysis based on upregulated genes in PDAC-A and PDAC-B cells identified in 0.7 kPa versus 38 kPa conditions. Individual genes important to enrichment of these functional nodes are shown. Colours represent genes upregulated in either PDAC-A (red), PDAC-B (blue) or both cell lines (split). PPAR, peroxisome proliferator-activated receptor. (F) Gene set enrichment plots showing enrichment of a matrisome gene set in ranked gene lists of both PDAC-A (top) and PDAC-B (bottom) cell lines. Genes were ranked based on signed −log_10_(*P*-value), 0.7 kPa versus 38 kPa. Red denotes relative enrichment and blue denotes relative depletion. NES, normalised enrichment score. *P*-values calculated using a phenotype-based permutation test.

To understand how pancreatic cancer cells respond to substrate stiffness, we cultured two independent PDAC cell lines, PDAC-A and PDAC-B, from KPC mice (*Pdx1*-Cre; LSL-*Kras*^G12D/+^; LSL-*Trp53*^R172H/+^; Morton et al., 2010), on fibronectin-coated polyacrylamide hydrogels of three defined stiffnesses, as reported previously (Papalazarou et al., 2020), and investigated gene expression profiles by performing RNA sequencing (Fig. 1A). We chose 0.7 kPa, 7 kPa and 38 kPa to model soft, intermediate and stiff tissue contexts respectively (Butcher et al., 2009), and included a glass coverslip condition (∼2–4 GPa) as a reference for typical cell culture conditions. As the stiffness of healthy pancreas is typically less than 1 kPa and PDAC tissue can reach 7–10 kPa, these stiffness values have physiological relevance to PDAC (Rice et al., 2017). Immunofluorescence imaging revealed clear morphological differences between different conditions, with cells on soft substrate appearing round and clustered compared to the elongated cells with visible protrusions and stress fibres seen on stiff substrate and glass (Fig. 1B). Furthermore, we observed a dramatic redistribution of the mechanosensitive transcriptional regulator YAP (Zanconato et al., 2019) from the nucleus to the cytoplasm on soft substrate (Fig. S1A,B), indicating that the system is indeed driving mechanosensitive pathways in PDAC cells.

To better understand the global changes in gene expression caused by different substrate stiffnesses, we conducted RNA sequencing of PDAC-A and PDAC-B cell lines on soft and stiff hydrogels, and glass. Hierarchical clustering of replicates revealed that the two cell lines had markedly distinct transcriptional profiles regardless of ECM stiffness (Fig. S1C), but that within cell lines replicates from soft conditions tended to cluster separately (Fig. S1D). Indeed, analysis of differentially expressed genes showed substantial changes in gene expression between the soft substrate condition and both stiff hydrogels and glass conditions (Fig. 1C,D). The observation that gene expression changes are consistent between stiff and glass conditions supports the idea that PDAC cells are mechanosensitive within a physiological range of substrate stiffnesses. Pathway analysis of genes upregulated on soft substrates revealed a surprising enrichment in genes associated with the ECM and substrate-interacting pathways (Fig. 1E). Gene-level analysis of the ‘ECM–receptor interaction’ pathway showed that a mix of ECM molecules (collagens, fibronectin) and integrins were upregulated on soft substrates (Fig. S1E). To confirm this, a gene set enrichment analysis between soft and stiff conditions was performed using the matrisome gene set from Naba et al. (2012). Both cell lines showed a significant enrichment in matrisome gene expression on soft hydrogels (Fig. 1F). Thus, PDAC cells show an orchestrated response to changes in mechanical stiffness of substrate characterised by increased expression of selected ECM-related genes.

### Collagen VI is upregulated by pancreatic cancer cells on soft substrates downstream of ECM adhesion and YAP

**Fig. 2.**
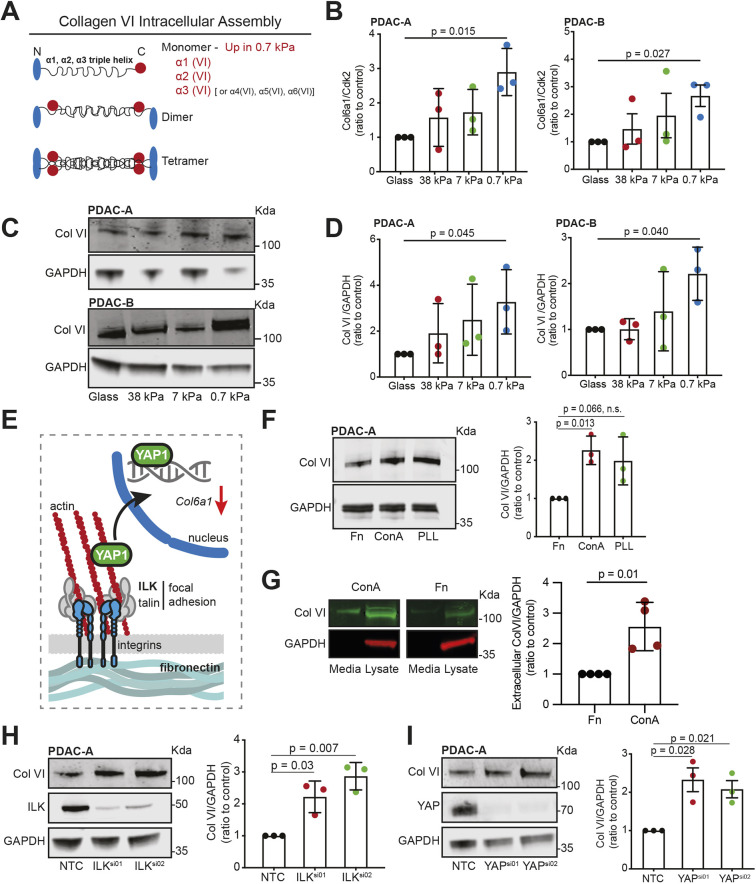
**Collagen VI is upregulated in PDAC cells upon low substrate stiffness.** (A) Schematic representation of the collagen VI assembly process in cells. The collagen VI monomer is a triple helix encoded by three genes: *Col6a1*, *Col6a2* and *Col6a3*. In certain cases, the chain encoded by *Col6a3* can be replaced by one encoded by *Col6a4* (not functional in humans), *Col6a5* or *Col6a6*. (B–D) PDAC-A and PDAC-B cells were cultured on 0.7 kPa, 7 kPa or 38 kPa fibronectin-coated hydrogels and glass coverslips, as indicated. (B) *Col6a1* mRNA expression was measured using RT-qPCR. Values are mean±s.d. relative to control expression (*Cdk2*) from three independent experiments. (C) Collagen VI protein expression was measured by immunoblotting for collagen VI (Col VI) and GAPDH (loading control). Blots are representative of three independent experiments. Position of molecular mass markers (in kDa) is indicated. (D) Densitometric quantification of protein for experiments as shown in C. Values are mean±s.d. of three independent experiments. (E) Schematic representation of main adhesion-linked pathways involved in sensing and responding to substrate stiffness by pancreatic cancer cells. (F) Left: PDAC-A cells were cultured on glass coverslips coated with fibronectin (Fn, control), concanavalin A (ConA) or poly-L-lysine (PLL), and immunoblotting for collagen VI and GAPDH (loading control) was performed. Right: densitometric quantification of collagen VI protein expression. Values are mean±s.d. of three independent experiments. (G) Left: immunoblots show the expression levels of collagen VI in the medium and cell lysates of PDAC-A cells cultured on concanavalin A (ConA)- or fibronectin (Fn, control)-coated plates, and immunoblotting for collagen VI and GAPDH (loading control) was performed. Right: densitometric quantification of extracellular collagen VI. Values are mean±s.d. of four independent experiments. (H) Left: control (NTC, non-targeting control) or ILK silenced (ILK^si01^ and ILK^si02^) PDAC-A cells were immunoblotted for collagen VI, ILK and α-tubulin (loading control). Right: densitometric quantification of collagen VI protein expression. Values are mean±s.d. of three independent experiments. (I) Left: control (NTC) or YAP silenced (YAP^si01^, YAP^si02^) PDAC-A cells were immunoblotted for collagen VI, YAP and GAPDH (loading control). Right: densitometric quantification of collagen VI protein expression. Values are mean±s.d. of three independent experiments. In B, D and F–I, statistical significance was assessed by two-tailed one-sample *t*-test on natural log-transformed values (n.s., not significant).

Upon comparing the gene-level changes in PDAC-A and PDAC-B cell lines, we noticed that several subunits of collagen VI had upregulated expression on soft matrix in both cell lines (Fig. S1E). *Ctgf* (also known as *Ccn2*), a well-described direct transcriptional target of YAP signalling, was downregulated on soft matrix in both cell lines, confirming that gene expression in these cancer cell lines is influenced by mechanosensing (Fig. S1F). In low-stiffness conditions, expression of *Fn1* (which encodes fibronectin), and of *Col6a1*, *Col6a2* and *Col6a3* (which encode collagen VI α1, α2 and α3 subunits, respectively), appeared upregulated on soft matrix in both cell lines. Other ECM-related genes, including genes encoding several subunits for collagens I–V, appeared unaffected by matrix stiffness (Fig. S1F), suggesting not a generalised typical fibrotic response, but rather a non-canonical upregulation of selected ECM components as a result of negative mechanosensing. Collagen VI is a fibrillar collagen that exists as a tetrameric assembly of α1 and α2 subunits, with possible inclusion of an α3, α4, α5 or α6 subunit (Fig. 2A). We first sought to confirm upregulation of collagen VI on soft substrates at the RNA and protein level. RT-qPCR of *Col6a1* revealed a gradual increase in mRNA levels across progressively softer substrates, which was significant when comparing the 0.7 kPa hydrogel and glass conditions (Fig. 2B). Increases in mRNA were shown to translate to protein expression via western blotting for collagen VI in both cell lines (Fig. 2C,D) as well as in PANC-1 human pancreatic cancer cells (Fig. S3A). Again, a gradual increase in protein expression was observed across the substrate stiffnesses, highlighting the sensitivity of collagen VI expression in PDAC cells to physical properties of the substrate. Imaging of collagen VI in PDAC-A and a separate human PDAC cell line (PANC1) showed a clear increase in intracellular collagen VI staining as substrate stiffness was reduced (Fig. S2A,B). Finally, to assess whether collagen VI expression could be an adaptive response to slower proliferation of PDAC cells upon low substrate stiffness (Papalazarou et al., 2020), we treated cells with aphidicolin, an inhibitor of DNA replication (Fig. S3B). Upon aphidicolin treatment there was a trend for reduced collagen VI expression, suggesting that the increase in collagen VI expression upon soft matrices is not a direct adaptation in response to lower proliferative capacity on soft matrix.

To better understand the mechanism driving increased expression of collagen VI on soft hydrogels, we conducted a range of experiments disrupting key steps in the integrin- and YAP-linked mechanosensation pathway (Fig. 2E). Firstly, to assess the requirement for integrin receptor engagement, PDAC-A cells were cultured on plastic coated with fibronectin, or on two substrates known to allow cell attachment without engagement of integrin receptors: concanavalin A (ConA) and poly-L-lysine (PLL) (Fig. 2F). Collagen VI expression was upregulated in both PDAC-A cells grown on ConA and those grown on PLL, suggesting that loss of integrin receptor engagement drives collagen VI expression. Moreover, this increased expression of collagen VI by cells on ConA-coated plates was followed by increased secretion of collagen VI into the culture medium (Fig. 2G), suggesting that collagen VI is not only accumulated intracellularly, but also deposited into the extracellular milieu. Integrin-linked kinase (ILK) is a component of focal adhesion complexes that translate integrin engagement to intracellular signalling (Hannigan et al., 2005). Transfection of PDAC-A cells with two different siRNAs targeting *Ilk* led to a 2–3-fold increase in collagen VI expression (Fig. 2H), and this effect was also observed in PDAC-B cells (Fig. S3C). Overexpression of a dominant-negative talin head domain mutant that disrupts binding to integrins (L325R) also resulted in increased collagen VI expression compared to levels in cells overexpressing a wild-type talin head domain (Fig. S3D,F). We also expressed the vinculin head domain (VD1) – which exerts a dominant-negative effect over endogenous vinculin, destabilising the connection of integrin–talin complexes to actin cytoskeleton – in PDAC-A cells. VD1 overexpression resulted in lower collagen VI expression (Fig. S3E,F), suggesting that it is not disruption of the talin-to-actin connection that results in increased collagen VI expression, but rather dysregulation of the integrin-to-talin part of mechanosensing. Finally, we asked whether collagen VI expression was directly altered by disruption of YAP, a major mechanosensitive transcriptional regulator that lies downstream of integrin–focal adhesion complex signalling. Silencing YAP expression using two siRNAs led to ∼2-fold increase in collagen VI expression (Fig. 2I). These results were confirmed using a CRISPR knockout (KO) of *Yap1* in PDAC-A cells, additionally showing that collagen VI expression was higher in *Yap1* KO cells regardless of substrate stiffness (Fig. S3G,H). Taken together, these results show that upregulation of collagen VI expression on soft substrates is likely driven by loss of integrin receptor engagement and subsequent YAP activity.

### Collagen VI supports focal adhesion turnover and invasive behaviours in PDAC cells

**Fig. 3.**
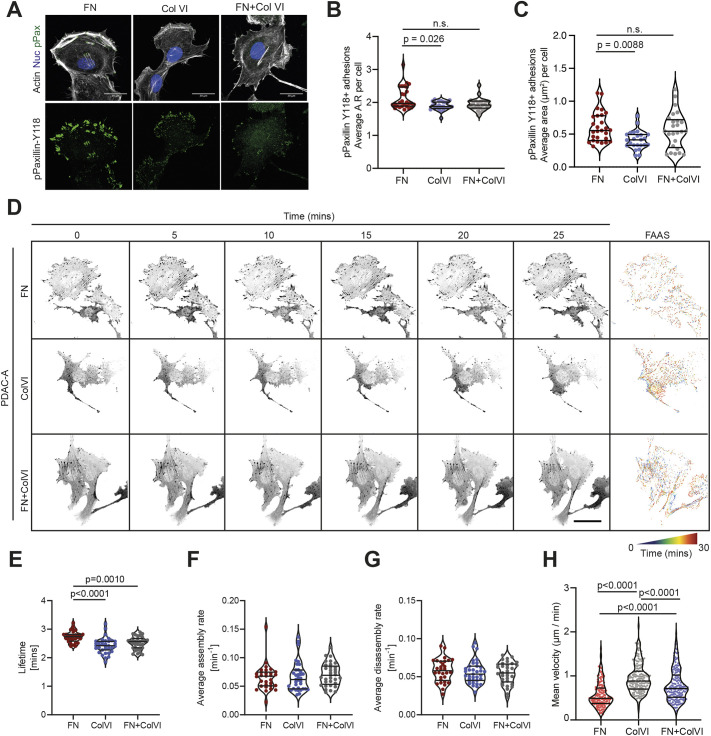
**Collagen VI ECM supports faster focal adhesion turnover and migratory behaviour of PDAC cells *in vitro*.** (A) Top: immunofluorescence of PDAC-A cells cultured on glass coverslips coated with fibronectin (FN), collagen VI (ColVI), or fibronectin and collagen VI (FN+ColVI), showing F-actin (phalloidin, grey), phospho-paxillin^­^ (pPaxillin-Y118; green) and nuclei (DAPI, blue). Bottom: individual pPaxillin-Y118 channel (green). Scale bars: 20 μm. (B,C) Quantification of pPaxillin-Y118-positive particles from A, showing focal adhesion average aspect ratio (AR) per cell (B) and average area (μm^2^) per cell (C). FN, *n*=27 cells; ColVI, *n*=24 cells; Fn+ColVI, *n*=25 cells. Cells are from three independent experiments. Statistical significance was assessed by Kruskal–Wallis test with Dunn's multiple comparisons test (B) and ordinary one-way ANOVA with Šídák's multiple comparisons test (C). n.s., not significant. (D–G) PDAC-A cells expressing paxillin–pEGFP were plated on fibronectin (FN), collagen VI (ColVI) or fibronectin and collagen VI (FN+ColVI). Images were acquired every minute for a total of 30 min. Focal adhesion turnover was assessed using the focal adhesion analysis server (FAAS). (D) Representative pictures of paxillin-positive focal adhesions (inverse greyscale) over time. Right-hand panel: FAAS output turnover image. Scale represents focal adhesion changes from blue to red over time. Scale bar: 50 µm. FAAS output of adhesion lifetime (E), assembly rates (F) and disassembly rates (G) for PDAC-A cells cultured on the different substrates as indicated. Statistical significance was assessed using a one-way ANOVA with Tukey's multiple comparisons test. Data from four independent experiments. FN, *n*=30 cells; ColVI, *n*=35 cells; FN+ColVI, *n*=33 cells. (H) Cell speed of PDAC-A cells plated on fibronectin (FN), collagen VI (ColVI) or fibronectin and collagen VI (FN+ColVI). Associated tracks are shown in Fig. S4B. FN, *n*=155 cells; ColVI, *n*=163 cells; Fn+ColVI, *n*=166 cells. Cells are from three independent experiments. Statistical significance was assessed by Kruskal–Wallis test with Dunn's multiple comparisons test. Violin plots in B, C and E–H display the median and quartiles.

Previous studies have found a role for adipose tissue-associated collagen VI in supporting breast cancer cell migration and invasion, although these studies did not investigate the influence of cancer cell-derived collagen VI (Iyengar et al., 2005; Wishart et al., 2020). To ask whether collagen VI alters pancreatic cancer cell morphology and migration, we cultured PDAC-A cells on either fibronectin, collagen VI or a 50:50 mix of the two (Fig. 3; Fig. S4A,B). Cells plated on collagen VI spread similarly (Fig. S4A) but produced significantly fewer mature focal adhesions, as marked by phosphorylated paxillin (Y118), indicating a less stable association with the substrate (Fig. 3B,C). To confirm this observation, we measured focal adhesion dynamics over time, finding that focal adhesions formed on substrates containing collagen VI had a shorter lifetime (Fig. 3D–G). Supporting this, timelapse imaging over a 16-h period revealed ∼2-fold increase in cell migration speed on collagen VI (Fig. 3H; Fig. S4B).

**Fig. 4.**
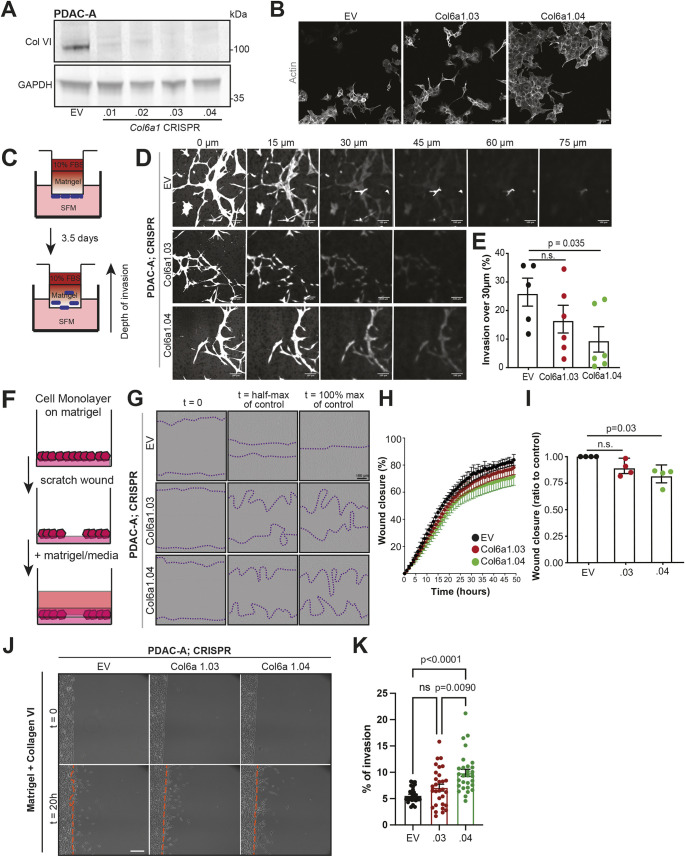
**Collagen VI supports invasion through recombinant basement membrane ECM.** (A) Control (EV) or collagen VI-depleted (Col6a1.01–Col6a1.04) mouse PDAC-A cell lines were immunoblotted for collagen VI and GAPDH (loading control). Blots shown are representative of three independent experiments. (B) Immunofluorescence of control (EV) or collagen VI-depleted (Col6a1.03 and Col6a1.04) PDAC-A cell lines showing actin (phalloidin, grey). Scale bars: 50 μm. Images are representative of three experiments. (C) Schematic representation of the inverted invasion assay setup. SFM, serum-free medium. (D) Representative pictures from *z*-stack acquisitions of control (EV) or collagen VI-depleted (Col6a1.03 and Col6a1.04) PDAC-A cell lines invading Matrigel plugs, showing Calcein AM staining after 72 h of invasion. Scale bars: 100 μm. (E) Quantification of percentage of cells invading further than 30 μm in the experiment described in D. Values are mean±s.e.m. from three independent experiments. Statistical significance assessed with two-tailed Welch's *t*-test (n.s., not significant). (F) Schematic representation of the 3D ECM wound invasion assay setup. (G) Representative pictures of control (EV) or collagen VI-depleted (Col6a1.03 and Col6a1.04) PDAC-A cells invading 3D ECM at the indicated times. Dotted lines indicate the extent of wound invasion. Scale bar: 100 μm. (H) Wound closure over time of control (EV) or collagen VI-depleted (Col6a1.03 and Col6a1.04) PDAC-A cells invading 3D ECM. Values are mean±s.d. from four independent experiments. (I) Relative wound closure calculated using data shown in H, normalised at *t*_1/2_ wound closure of the control. Values are mean±s.d. from four independent experiments. Statistical significance assessed by two-tailed one-sample *t*-test on natural log-transformed values. (J) Invasion assay showing representative images of control (EV), and collagen VI-depleted PDAC-A cells (Col6a1.03 and Col6a1.04) at *t*=0 h and *t*=20 h after invading Matrigel containing collagen VI. Scale bar: 200 μm. (K) Quantification of percentage of invasion for the experiment described in J. Mean±s.e.m. from *n*=3 independent experiments, each with *n*>10 fields of view per condition quantified. Statistical significance assessed by Kruskal–Wallis test with Dunn's multiple comparisons test (ns, not significant).

As these experiments utilised exogenous recombinant protein, it is unclear whether cancer cell-derived collagen VI can support migratory behaviour in the same manner. To test this, we selected two of four PDAC-A CRISPR lines (Col6a1.03 and Col6a1.04) in which a critical exon of *Col6a1* was deleted (Fig. 4A). These cells appeared morphologically normal, but we did observe a slight increase in cell area in the Col6a1.03 line (Fig. 4B; Fig. S4C). We used two complementary experiments to investigate the invasive potential of these cells. Firstly, invasion into a Matrigel plug in response to a serum chemotactic gradient was used (Fig. 4C). Analysis of the depth that cells invaded into the plug after 72 h revealed a reduction that was significant for Col6a1.04 (Fig. 4D,E; Fig. S4D). Secondly, we utilised a 3D wound healing assay in which a Matrigel-embedded cell monolayer invades into wounded ECM (Fig. 4F). We again observed a reduction in wound closure over a 50-h period that was significant for the Col6a1.04 line (Fig. 4G–I). In addition, offering extracellular collagen VI in Matrigel plugs rescued the ability of Col6a1-depleted cancer cells to invade within Matrigel (Fig. 4J,K). Collagen VI KO cells proliferated at similar rates *in vitro* compared to empty vector (EV) controls (Fig. S4E). Taken together, these results indicate that cell-derived collagen VI in pancreatic cancer cells supports invasive behaviours in a range of contexts.

### Collagen VI deposition increases during PDAC progression and is associated with poor survival

**Fig. 5.**
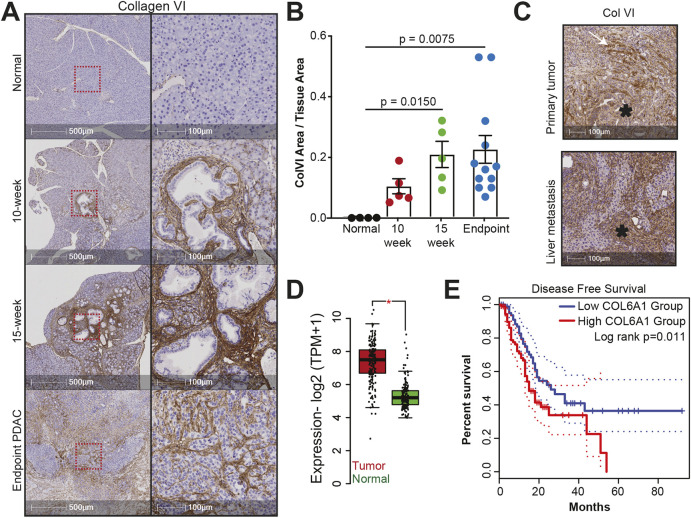
**Collagen VI is upregulated during PDAC progression and correlates with reduction in disease-free survival**. (A) Immunohistochemistry of normal mouse pancreas (top panel) and of 10-week, 15-week and endpoint PDAC pancreas from KPC mice showing collagen VI expression. Samples are counterstained with Haematoxylin Z. Boxes in the left-hand images indicate magnified areas shown on the right. Scale bars: 500 μm (left), 100 μm (right). (B) Quantification of collagen VI-positive area per tissue area for the experiment described in A. Values are mean±s.e.m. Normal, *n*=4 mice; 10-week, *n*=5 mice, 15-week, *n*=5 mice; and endpoint, *n*=12 mice. Statistical significance assessed by Kruskal–Wallis test with Dunn's multiple comparisons test. (C) Representative images of immunohistochemistry from endpoint PDAC pancreas (primary tumour) and metastatic nodules in the liver from KPC mice, showing collagen VI expression. Samples are counterstained with Haematoxylin Z. Asterisks indicate pancreatic tumour cells. White arrow indicates collagen VI-positive fibres. Images are representative of *n*=3 mice with matched primary tumour and liver metastasis samples. Scale bars: 100 μm. (D) Expression of *COL6A1* is upregulated in the tumours of pancreatic cancer patients compared to normal pancreas tissue specimens. Data taken from TCGA and the Genotype–Tissue Expression project (GTEx). Tumour, *n*=179 patients; normal: *n*=171 patients. Boxplots show median (bar), interquartile range (box) and 1.5× interquartile range (whiskers). Statistical significance assessed by two-tailed two-sample *t*-test.*P≤0.05. TPM, transcripts per million. (E) Kaplan–Meier plot of disease-free survival, stratifying patients based on *COL6A1* expression in pancreatic tumours. High *COL6A1* expression is associated with a significant reduction in disease free survival (log rank *P*<0.012). *n*=89 patients with high *COL6A1* expression and *n*=89 patients with low COL6A1 expression. Dotted lines indicate 95% confidence intervals.

Having shown that pancreatic cancer cells display mechanosensitive expression of collagen VI, and that collagen VI supports their motility in 2D and 3D settings, we investigated whether cancer cell-derived collagen VI supports pancreatic cancer progression *in vivo*. Collagen VI has well documented roles in certain cancers including breast (Wishart et al., 2020), ovarian (Sherman-Baust et al., 2003) and lung (Voiles et al., 2014). We began by investigating whether collagen VI expression is altered in the KPC mouse model of PDAC. We stained pancreata of normal mice (*Pdx1*-Cre; *Kras*^+/+^; *Trp53*^+/+^) and of KPC mice (*Pdx1*-Cre; *Kras*^G12D/+^; *Trp53*^R172H/+^) at different stages of PDAC progression from pancreatic intraepithelial neoplasia (PanIN) I–III (10- and 15-week) to endpoint PDAC (Fig. 5A). We noticed an upregulation of collagen VI expression with PDAC progression (Fig. 5B). Furthermore, collagen VI was mainly localised in the stroma, suggesting that stromal cells (particularly CAFs) may be the primary depositors of this type of collagen VI in primary PDAC tissue, which is in agreement with a recent proteomic study (Tian et al., 2019). As primary PDAC tissue is typically stiff, it is unsurprising that cancer cells are not the major source of collagen VI in primary tissue. Indeed, imaging of collagen VI expression in liver metastasis samples of KPC mice (where cells will be experiencing softer substrate) revealed markedly stronger staining that also appeared to be more cytoplasmic and PDAC cell derived (Fig. 5C).

To confirm that these results were representative of human disease, we consulted data from The Cancer Genome Atlas (TCGA) database of primary human PDAC tissue (Fig. 5D,E). RNA levels of *COL6A1* were significantly increased in primary tumour samples versus levels in normal pancreas samples (Fig. 5D). Furthermore, high *COL6A1* expression was associated with reduced disease-free survival in these PDAC patients (Fig. 5E). TCGA expression datasets are derived from multiple cell types, including cancer cells and a heterogeneous mixture of stromal cells. Thus, it is possible that collagen VI extracellular deposition in primary PDAC tissue may be driven by stromal cells – either CAFs or activated immune cells. As mentioned above, collagen VI staining in primary PDAC seems to be localised in the stroma, and our results suggests that pancreatic cancer cell-specific collagen VI expression may be important primarily for metastatic seeding.

### Collagen VI expression supports establishment of pancreatic metastasis *in vivo*

**Fig. 6.**
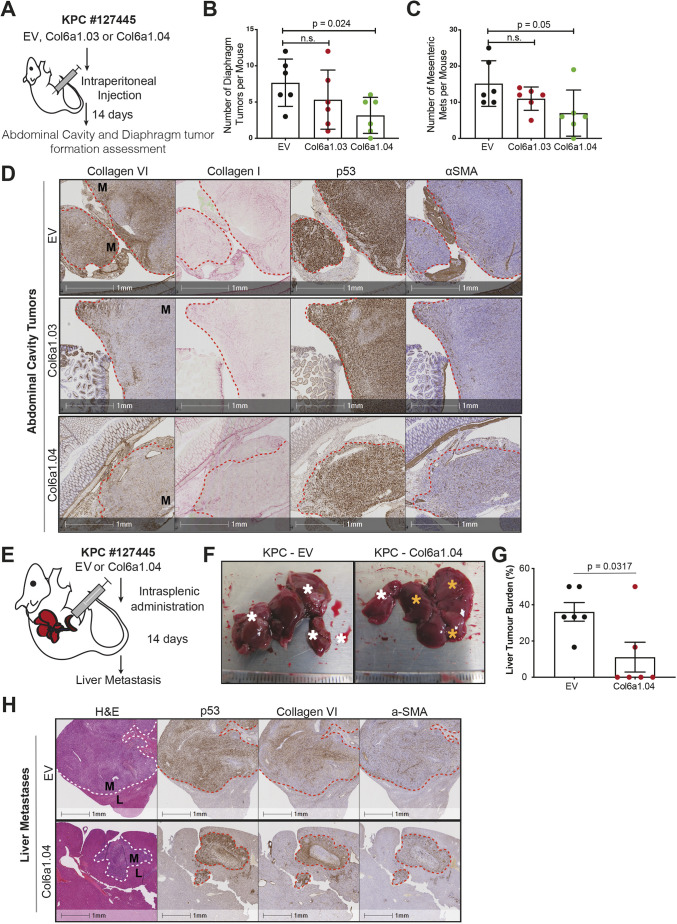
**Collagen VI expression supports establishment of pancreatic metastasis *in vivo*.** (A) Schematic of IP injection model. Control (EV) or collagen VI-depleted (Col6a1.03 and Col6a1.04) KPC #127445 cells were injected in the IP cavity of nude (Cd1-nu) mice. After 14 days, the mice were euthanised, and the number of tumours was quantified before fixation of isolated tissues. (B) Quantification of diaphragm tumours per mouse following IP injection as indicated. Values are mean±s.d. EV, *n*=6 mice; Col6a1.03, *n*=6 mice; and Col6a1.04, *n*=6 mice. Statistical significance was assessed with a two-tailed Welch's *t*-test (n.s., not significant). (C) Quantification of abdominal cavity tumours per mouse following IP injection as indicated (mesenteric mets, mesenteric metastases). Values are mean±s.d. EV, *n*=6 mice; Col6a1.03, *n*=6 mice; and Col6a1.04, *n*=6 mice. Statistical significance assessed with a two-tailed Welch's *t*-test. (D) Representative immunohistochemistry images showing collagen VI, collagen I, p53 (marks tumour, with mutant p53 accumulation) and αSMA (marks fibroblasts) expression in abdominal cavity tumour nodules formed by IP injection of control (EV) or collagen VI-depleted (Col6a1.03 and Col6a1.04) KPC #127445 cells. Red dashed line denotes boundary between normal tissue and metastases (M). Images are representative of tissue stained from *n*=6 mice per condition. (E) Schematic of IS injection model. Control (EV) or collagen VI-depleted (Col6a1.04) KPC #127445 cells were transplanted into the spleen of nude (CD1-nu) mice and 14 days later formation of metastatic tumours in the liver was assessed. (F) Representative liver pictures at the time of dissection. White asterisks indicate lobes with metastatic tumours and yellow asterisks indicate tumour-free liver areas. Scale graduations are in mm. (G) Liver tumour burden (% of liver lobes affected) of animals treated as indicated. Values are mean±s.e.m. from *n*=6 EV mice and *n*=6 Col6a1.04 mice. Statistical significance was assessed with a two-tailed Welch's *t*-test. (H) Representative immunohistochemistry images showing H&E staining and expression of p53, collagen VI and α-SMA (a-SMA) in liver sections from F, as indicated. Images are representative of tissue stained from *n*=6 mice per condition. Dashed lines indicate metastatic areas. L, liver; M, metastasis.

We hypothesised that PDAC cells might upregulate collagen VI upon extravasation in the liver, contributing to the establishment of the metastatic niche. To test this hypothesis, we initially performed intraperitoneal (IP) injection experiments using a KPC mouse-derived cell line (KPC #127445) that we have used previously for such experiments (Juin et al., 2019) (Fig. 6A). Two KPC #127445 CRISPR lines with KO of *Col6a1* (Col6a1.03 and Col6a1.04) were used alongside an EV control to generate abdominal cavity and diaphragm tumours (Fig. 6A; Fig. S5A). Interestingly, both *Col6a1* KO lines showed a trend for decreased tumour formation on the diaphragm (Fig. 6B) and the peritoneum (Fig. 6C), which was significant for the Col6a1.04 line, despite no changes in mouse weight (Fig. S5B). Histological analysis of these tumours confirmed the high levels of collagen VI expression in abdominal cavity tumours from EV cells and the significant loss of Col6a1 staining in both KO lines (Fig. 6D). What collagen VI staining remained in *Col6a1* KO tumours appeared to colocalise with αSMA (ACTA2)-positive cells, which likely represent myofibroblast-like resident cells (Fig. S5C). Although we cannot determine the origin of this surrounding collagen VI in Col6a1 KO tumours, the tumour-associated fibroblasts are the most likely source.

The liver is a common site of metastasis in pancreatic cancer. To further validate these observations, and since IP injections do not represent the metastatic cascade, we decided to assess the role of PDAC-derived collagen VI in formation of liver metastases using the model of intrasplenic (IS) transplantations (Fig. 6E). Interestingly, collagen VI KO cells (Col6a1.04) displayed a reduced ability to form metastases in the liver compared with EV control cells following IS transplantation (Fig. 6F,G). Histological staining again showed that collagen VI was deposited primarily in areas with high αSMA-positive cells in KO conditions and was more diffuse in EV control tumours (Fig. 6H). Furthermore, KO metastases typically had strong collagen VI deposition surrounding tumour nodules, likely of stromal origin, suggesting that presence of collagen VI is required to support metastasis formation, regardless of its source. In both IP and IS injection models, we didn't observe a proliferation disadvantage following collagen VI KO in these cancer cells, as measured by Ki-67 (also known as MKI67) positivity in metastatic foci (Fig. S5D,E). We validated these histological results by generating liver metastases with PDAC-B CRISPR control (EV) or Col6a1 KO (Col6a1.03) cell lines (Fig. S5F,G). Analysis of common ECM markers and stromal cell types showed no significant differences between the EV and Col6a1.03 conditions, although there was a trend for a 1.5–2-fold increase in abundance of fibroblasts (αSMA^+^), neutrophils (Ly6G^+^) and macrophages (F4/80^+^) in collagen VI KO tumours. Thus, while cancer cell-derived collagen VI could be important for the formation of metastases at multiple organ sites, other cell types in the metastatic microenvironment might also be able to deposit collagen IV, facilitating metastatic seeding.

## DISCUSSION

Pancreatic tumours are hallmarked by their stiff collagen-rich stroma. However, the metastatic cascade requires cells to adapt to softer environments, raising the question of how this plasticity is achieved. Culturing PDAC cells on hydrogels of varied stiffnesses showed substantial reprogramming of cells, which was accompanied by morphological changes that have been reported previously (Papalazarou et al., 2020). Despite having the same driver mutations (*Kras^G12D^* and *Trp53^R172H^*), the two PDAC cell lines isolated from KPC mice each showed unique responses to culture on soft versus stiff hydrogels, both at the individual gene expression level and the gene programme level. This reflects the heterogeneity of PDAC cell identity also observed in the clinical setting, which contributes to pancreatic cancer resistance to therapeutic intervention (Carstens et al., 2021). Given this, it is noteworthy that an upregulation of collagen VI, and of matrisome genes more broadly, was a shared response of both PDAC lines to culture on soft substrate. ECM secretion by cancer cells in low-adhesion environments has been suggested previously; for example, increased expression of SPARC, MPG and SPON2 has been reported in circulating tumour cells from patients (Ting et al., 2014). However, direct evidence for specific stiffness-dependent expression of ECM proteins by PDAC cells, as presented here, has been lacking. Our data adds to a growing repertoire of mechanosensitive mechanisms that PDAC cells use to survive and grow in different niches.

Our data from experiments disrupting various components of the mechanosensation pathway through plating cells on substrates that do not engage integrins, disruption of vinculin or talin interactions, or knockdown of ILK (Fig. 2) strongly suggest that increased collagen VI expression on soft substrates is due to a lack of integrin receptor engagement and downstream YAP nuclear translocation. How a reduction in YAP-driven transcription leads to increased collagen VI transcription is currently unclear. YAP coordinates expression of large gene networks, including those that can actively repress expression of other genes (Kim et al., 2015). Thus, increased collagen VI expression on soft substrates could be a consequence of relieved inhibition of another factor. Alternatively, inactivation of YAP might free TEAD transcription factors to interact with other co-factor transcription factors, such as VGLL proteins (Zhang et al., 2017), to promote expression. The differing responses of PDAC-A and PDAC-B cells to soft hydrogels highlight the complexity of mechanosensitive expression and suggest that the exact pathways activated may differ depending on other properties or cell states.

An increasingly important question in relation to cancer ECM is the cellular origin and mechanism of action of specific ECM components. Along with most collagens, collagen VI expression is upregulated in primary PDAC and mostly derives from myofibroblasts (Tian et al., 2019). This is supported by data here showing that PDAC cells downregulate their own expression of collagen VI in a stiff environment such as primary PDAC tumours. Collagen VI secretion in primary PDAC could be the result of the general desmoplastic response occurring in tumours of pancreatic origin and is mainly driven by stromal cells. Stromal cells might have their own distinct regulation for collagen VI expression, with the latter being an integral component of an extended network of stromal cell-derived extracellular proteins in primary tumours. Stroma-derived collagen VI may still play important roles in PDAC progression, such as promoting motility, as has been reported for breast cancer (Wishart et al., 2020). Indeed, our data showing increased motility of PDAC cells on collagen VI-coated coverslips support this. Comparing metastatic tumours formed by PDAC cells with or without KO of *Col6a1*, it is clear that cancer cells are a significant source of collagen VI in this context. Furthermore, there is a heterogeneity of stiffness across PDAC tumours, with some areas showing higher stiffness than others (Rice et al., 2017). It is intriguing to hypothesise that cancer cells from areas with low stiffness have increased collagen VI expression and therefore have an advantage in establishing metastasis. Future studies could explore the metastatic potential of cancer cells derived from low-stiffness areas versus that of cells from high-stiffness areas. Nevertheless, cancer cell-derived ECM factors emerge as important enablers of metastatic seeding. Recently, it has been shown that dormant cancer cells are able to secrete and establish a type III collagen-enriched ECM niche that maintains their dormant phenotype. Type III collagen induces dormancy and sustains tumour growth in primary tumours, suggesting therapeutic potential in using ECM components to direct cancer cell fate (Di Martino et al., 2022). Therefore, investigating how the different ECM components influence cancer cell status in metastatic dissemination and which mechanisms are involved in their deposition arise as important questions for future studies.

Our data show that cancer cell-derived collagen VI has cell autonomous roles in determining the metastatic potential of PDAC cells. Using a number of approaches, we show that collagen VI supports invasive potential of PDAC cells *in vitro* and *in vivo*. This aligns with other recent studies reporting promotion of migration and invasion by collagen VI in other cancers (Chen et al., 2013; Wishart et al., 2020). Given that we see enhanced migration of PDAC cells on collagen VI-coated coverslips, our data support a mechanism whereby motility effects are due to secreted collagen VI providing a local substrate for cancer cells. However, we cannot discount the possibility that collagen VI may have additional intracellular functions or that collagen VI may accumulate in cells on soft substrates due to an inability to be secreted. Additionally, the COL6A3 fragment endotrophin (ETP) has been suggested to enhance EMT in cancer cells (Park and Scherer, 2012), although it is not responsible for migration effects in breast cancer cells (Wishart et al., 2020). Finally, collagen VI may also support establishment of metastases by providing ECM peptides for attachment to the liver vasculature during early seeding, as has been shown for PDAC cell-derived matrisome proteins SERPINB5 and CSTB (Tian et al., 2020).

In summary, this study uncovers a mechanosensitive mechanism whereby pancreatic cancer cells alter their own ECM environment to support metastatic colonisation. Using a combination of gene expression profiling, *in vitro* characterisation and *in vivo* data, we show how collagen VI expression is dependent on substrate stiffness. Our *in vivo* data also suggest that collagen VI is an important factor in the liver metastatic niche and can be provided either by the tumour cells or the liver resident cells. Our study highlights the dual nature of mechanosensation – as a response to soft as well as stiff environments – and illustrates the importance of the former to metastatic dissemination.

## MATERIALS AND METHODS

### Cell culture

All cell lines used in this study were cultured at 37˚C under 5% CO_2_ in a humidified incubator and were tested regularly for mycoplasma contamination. Primary murine (PDAC-A and PDAC-B) and human PANC-1 cells were cultured in high-glucose DMEM (Gibco, 21969-035) supplemented with FCS (10%; Gibco, 10270-106), glutamine (2 mM), sodium pyruvate (0.11 g l^−1^), penicillin (10,000 units ml^−1^) and streptomycin (10,000 units ml^−1^). PDAC-A and PDAC-B cell lines were a gift from Jennifer Morton and Saadia Karim (CRUK Beatson Institute, Glasgow, UK), and were isolated from the tumours of *Pdx1*-Cre; LSL-*Kras*^G12D/+^; LSL-*Trp53*^R172H/+^ (KPC) mice with a mixed background. Human PANC-1 cells were from ATCC (CRL-1469) but were not independently authenticated.

### Reagents and oligonucleotides

The following reagents were used: human fibronectin protein (1918-FN-02M, R&D Systems); fibronectin bovine plasma (F1141, Sigma-Aldrich); concanavalin A from *Canavalia ensiformis* (L7647, Sigma-Aldrich); poly-L-lysine (P4707, Sigma-Aldrich); aphidicolin from *Nigrospora sphaerica* (A4487, Sigma-Aldrich); Corning Matrigel Basement Membrane Matrix (354234, Corning); rat tail collagen I (354249, Corning); Sigmacote (SL2, Sigma-Aldrich); sulfosuccinimidyl 6-(4′-azido-2′-nitrophenylamino)hexanoate (sulfo-SANPAH; 22589, Thermo Fisher Scientific); 3-(acryloyloxy)propyltrimethoxysilane (L16400, Alfa Aesar); acrylamide, 40% solution (A4058, Sigma-Aldrich); N,N′-methylene-bis-acrylamide, 2% solution (M1533, Sigma-Aldrich); N,N,N′,N′-tetramethylethylenediamine (TEMED; T9281, Sigma-Aldrich); ammonium persulfate (A3678, Sigma-Aldrich); ammonium hydroxide (221228, Sigma-Aldrich); hydrogen peroxide (30% w/w; 31642, Sigma-Aldrich); puromycin dihydrochloride (A1113803, Thermo Fisher Scientific); Lipofectamine RNAiMAX (13778150, Thermo Fisher Scientific); Lipofectamine 2000 (11668019, Thermo Fisher Scientific); Lullaby (FLL73000, OZ Biosciences); Amaxa Cell Line Nucleofector Kit V (VCA-1003, Lonza); Precision Red Advanced Protein Assay (ADV02-A; Cytoskeleton); and Calcein AM (C1430, Thermo Fisher Scientific).

For CRISPR/Cas9-mediated genome editing, the following sequences were cloned into pSpCas9(BB)-2A-Puro (PX459) V2.0 (Addgene 62988, deposited by Feng Zhang, Massachusetts Institute of Technology, Cambridge, MA, USA): 5′-GTACTTGACCGCATCCACGC-3′ (Col6a1.01), 5′-TTGAGCTCATCGCGGCCAC-3′ (Col6a1.02), 5′-CTTGATCGTGGTGACCGAC-3′ (Col6a1.03) and 5′-ACTTGATCGTGGTGACCGA-3′ (Col6a1.04) targeting mouse *Col6a1*; and 5′-ACTTGATCGTGGTGACCGA-3′ (YAP.01) targeting mouse *Yap1*. For siRNA-mediated silencing the following sequences were used: 5′-ACCAGGTCGTGCACGTCCGC-3′ (YAP^si01^) and 5′-ATGGAGAAGTTTACTACATAA-3′ (YAP^si02^) targeting mouse *Yap1*, and 5′-CCGCAGTGTAATGATCGATGA-3′ (ILK^si01^) and 5′-CTCTACAATGTTCTACATGAA-3′ (ILK^si02^) targeting mouse *Ilk*.

The following DNA constructs were used: pEFGP-C1 (host lab), pEGFPC1/GgVcl1-258 (VD1; Addgene 46270, deposited by Susan Craig, Johns Hopkins School of Medicine, Baltimore, MD, USA; Cohen et al., 2006), EGFP–talin1 head domain (Addgene 32856, deposited by Anna Huttenlocher, Harvard Medical School, Cambridge, MA, USA; Simonson et al., 2006), paxillin–pEGFP (Addgene 15233, deposited by Rick Horwitz, Allen Institute for Cell Science, Seattle, WA, USA; Laukaitis et al., 2001). The L325R mutation was introduced to the EGFP–talin1 head domain construct using the Q5 site-directed mutagenesis kit (NEB, E0554).

### Polyacrylamide hydrogel preparation

Polyacrylamide hydrogels were prepared at 0.7 kPa, 7 kPa and 38 kPa stiffness values as previously described (Papalazarou et al., 2020). Polyacrylamide hydrogels were treated with 0.2 mg ml^−1^ sulfo-SANPAH solution in Milli-Q water followed by UV irradiation (365 nm) for 10 min. Hydrogels were extensively washed with 50 mM HEPES buffer (pH 8.5), incubated overnight with fibronectin (10 μg ml^−1^) and washed extensively in PBS before use.

### ECM coatings

Glass coverslips were washed with ethanol, oven-dried and then coated either with fibronectin (10 μg ml^−1^) for 60 min at room temperature, or with concanavalin A (10 μg ml^−1^) or poly-L-lysine (0.5 mg ml^−1^) for 16 h at 4°C. Coated coverslips were washed in PBS, and cells (2×10^4^ cells per ml) were plated and cultured for 16 h in DMEM containing 10% FBS.

### RNA extraction, RNA sequencing and RT-qPCR

Total RNA extraction and purification from cells was performed using the RNeasy Mini Kit (Qiagen) combined with RNaseFree DNase (Qiagen) treatment, following the manufacturer's instructions. Measurements of RNA concentration and purity were routinely performed using a NanoDrop 2000C (Thermo Fisher Scientific) before downstream processing. For RNA sequencing, RNA quality was tested using the Agilent Technologies 2200 TapeStation instrument according to the manufacturer's instructions. Briefly, 5 μl of RNA sample buffer was mixed with 1 μl of RNA ladder and added to the first tube of a RNAse-free mini-tube strip. Then, 5 μl of RNA sample buffer was mixed with 1 μl of RNA sample and loaded into the strip. Samples were vortexed and centrifuged for 1 min at 300 ***g***. Samples were heated to 72°C for 3 min and then placed on ice for 2 min. Samples were centrifuged for 1 min at 300 ***g*** and then loaded into the Agilent 2200 TapeStation instrument.

Libraries were prepared using a Library Prep kit (Illumina TruSeq RNA Sample prep kit v2) and were run on an Illumina NextSeq500 platform using a High Output v2 75 cycles (2×36 cycle, paired end, single index) sequencing kit. Quality checks on the raw RNA sequencing data files were performed using FastQC (https://www.bioinformatics.babraham.ac.uk/projects/fastqc/) and FASTQ screen (http://www.bioinformatics.babraham.ac.uk/projects/fastq_screen/) tools. RNA sequencing reads were aligned to the GRCm38 version of the mouse genome using TopHat2 version 2.0.13 (Kim et al., 2013) with Bowtie version 2.2.4.0 (Langmead and Salzberg, 2012). Expression levels were determined and statistically analysed by a combination of HTSeq version 0.7.2 (http://www.huber.embl.de/users/anders/HTSeq/doc/overview.html; Putri et al., 2022), the R 3.3.3 environment – utilising packages from the Bioconductor data analysis suite – and differential gene expression analysis based on a generalised linear model using the DESeq2 package (Love et al., 2014). Significantly differentially expressed genes (*P*_adj_<0.05, DESeq2 Wald test) were submitted to DAVID (https://david.ncifcrf.gov/) for gene ontology (GO) analysis. Kyoto Encyclopedia of Genes and Genomes (KEGG) pathway analysis (https://www.genome.jp/kegg/pathway.html) was performed for genes demonstrating a greater than 1.5 log_2_ fold change increase or decrease in RNA expression between conditions. Significant KEGG GO Terms were identified (*P*_adj_<0.05, Wald test with Benjamini-Hochberg adjustment using clusterProfiler; Yu et al., 2012). Hierarchical clustering of log_2_ fold differentially expressed genes was performed on the basis of Euclidean distance using complete linkage and was visualised using the Rstudio, v.1.1.453 environment. Principal component analysis was performed using FactoMineR version 2.3 (Lê et al., 2008). We also performed DESeq2 analysis as well as category netplot (CNET) analysis using the Bioconductor data analysis suite and the Rstudio, v.1.1.453 environment. The matrisome gene set source was taken from https://www.gsea-msigdb.org/gsea/msigdb/cards/NABA_MATRISOME (accessed 02/2022) for gene set enrichment analysis using GSEABase (https://bioconductor.org/packages/release/bioc/html/GSEABase.html).

cDNA synthesis was performed using either a DyNAmo cDNA synthesis kit (F-470 L, Thermo Fisher Scientific) or a Maxima First Strand cDNA synthesis kit (K1641, Thermo Fisher Scientific). Then, qPCR was performed using the DyNAmo HS SYBR Green qPCR kit (F410 L, Thermo Fisher Scientific). PCR was performed on a C1000 Thermal Cycler (CFX96 Real time system, BioRad) as follows: 3 min at 95°C; 40 cycles of 20 s at 95°C, 30 s at 57°C and 30 s at 72°C; and a final 5 min at 72°C. Relative mRNA quantification was performed using the 2^−ΔΔCt^ method for multiple genes, with *Cdk2* used as the control gene. Primer sequences used were: Cdk2-Fw, 5′-TGAAATGCACCTAGTGTGTACC-3′; Cdk2-Rv, 5′-TCCTTGTGATGCAGCCACTT-3′; Col6a1-Fw, 5′-CGTGGAGAGAAGGGTTCCAG-3′; Col6a1-Rv, 5′-GTCTCTCCCTTCATGCCGTC-3′.

### Growth curves

To assay growth, 5×10^4^ cells were seeded on 12-well plates in triplicate wells and allowed to adhere overnight. The next day (t=0), the medium was renewed and cells were counted at 48 h and 96 h using a CASY Model TT cell counter (Innovatis, Roche Applied Science). The medium was renewed with fresh fully supplemented DMEM at 48 h.

### Western blotting

Proteins were isolated in radioimmunoprecipitation (RIPA) lysis buffer [150 mM NaCl, 10 mM Tris-HCl pH7.5, 1 mM ethylenediaminetetraacetic acid (EDTA), 1% Triton X-100, 0.1% SDS, 1× protease and phosphatase inhibitors (Roche)]. Protein concentration was determined using a Precision Red Advanced Protein Assay. Protein lysates were mixed with NuPAGE LDS Sample Buffer (Thermo Fisher Scientific, NP0007) and NuPAGE Sample Reducing Agent (Thermo Fisher Scientific, NP0004), and 20 μg protein was loaded onto 8–12% SDS-PAGE gels and transferred onto nitrocellulose membranes. For protein isolated from culture medium, the same number of cells (8×10^4^) was plated in each well of a 6-well dish coated with fibronectin or concanavalin A, and cells were starved of FBS for 16 h before lysis. Media were centrifuged at 3500 ***g*** for 10 min, and 1.8 ml of medium was transferred to a concentrating column (Amicon Ultra-4 centrifugal filter) for each sample. Following centrifugation at 3800 ***g*** for 10 min, 20 µl of 2× NuPAGE LDS Sample Buffer (Thermo Fisher Scientific, NP0007) and NuPAGE Sample Reducing Agent (Thermo Fisher Scientific, NP0004) was added to 20 μl of the concentrated medium, and the mixture was loaded onto 8–12% SDS-PAGE gels. Membranes were blocked (3% BSA in TBS containing 0.1% Tween-20) and incubated for 16 h in 4°C with one of the following antibodies: anti-collagen VI (1:1000; ab182744, EPR17072, Abcam, lot GR242914-18), anti-YAP (1:1000; 14074, clone D8H1X, Cell Signaling Technology, lot 4), anti-ILK (1:1000; 3856S, clone 4G9, Cell Signaling Technology, lot 3), anti-ERK1/2 (1:1000; 9102, clone L34F12, Cell Signaling Technology, lot 26), anti-GAPDH (1:1000; MAB374, clone 6C5, Millipore, lot 3249425) and anti-α-tubulin (1:3000; T6199, clone DM1A, Sigma-Aldrich, lot 029M4842V). Protein detection was achieved using Alexa Fluor-conjugated secondary antibodies, and signal was imaged using the LI-COR Odyssey CLx (LI-COR Biosciences) system. All images were analysed using Image Studio Lite software, version 5.2.5.

### Cell transfection and genetic modifications

Cells were typically transfected in suspension. 5 μg of DNA was used to transfect the cells using the Amaxa Cell Line Nucleofector kit (Lonza Bioscience) according to the manufacturer's instructions. To generate stable cell lines, stably transfected cells were selected with G418 (G418S, Formedium) followed by fluorescence activated cell sorting (FACS). For FACS, gating was performed by cell size, live/dead and GFP-positive signal.

For transfection of adhered cells, 1 μg of DNA was used with Lipofectamine 2000 reagent (Thermo Fisher Scientific) according to the manufacturer's instructions. For siRNA-mediated YAP silencing, *Yap1*-targeting siRNA oligonucleotides (25 nM; Qiagen) or control siRNA (25 nM; AllStars Negative Control siRNA, Qiagen) were used to transfect the cells using the Lipofectamine RNAiMAX transfection reagent (Thermo Fisher Scientific) according to the manufacturer's instructions. YAP-silenced cells were analysed 48 h post transfection. For siRNA-mediated ILK silencing, *Ilk-*targeting oligos (15 nM; Qiagen) or control siRNA (15 nM; AllStars Negative Control siRNA, Qiagen) were used with the Lullaby transfection reagent (OZ Biosciences) according to the manufacturer's instructions. Two-step siRNA delivery with 48 h interval was performed, and ILK-silenced cells were analysed 24 h post transfection. For CRISPR/Cas9-mediated KO, cells were transfected with 5 μg of selected plasmid (either control empty vector or vectors containing guide RNAs) using Amaxa Cell Line Nucleofector kit (Lonza Bioscience) according to the manufacturer's instructions. Stably transfected cells were selected using puromycin (2 μg ml^−1^).

### Immunofluorescence

Typically, 2×10^4^ cells per cm^2^ were seeded on 19-mm diameter coverslips or polyacrylamide hydrogels for 16 h. Cells were washed 1× in PBS and fixed in 4% paraformaldehyde for 10 min before being permeabilised with 0.1% Triton X-100 for 5 min followed by 30 min incubation in blocking buffer (1% BSA in PBS). Cells were incubated for 60 min with the following primary antibodies: anti-YAP (1:100; 14074, clone D8H1X, Cell Signaling Technology, lot 4), anti-phospho-paxillin (Y118) (1:400; 2541, Cell Signaling Technology, lot 6) and anti-collagen VI (1:200; ab182744, Abcam, lot GR242914-18). Detection was performed using the following secondary antibodies: Alexa Fluor 488-conjugated donkey anti-rabbit IgG (1:500; A21206, Invitrogen) and Alexa Fluor 594-conjugated donkey anti-mouse IgG (1:500; A21203, Invitrogen). Nuclei were visualised using DAPI (0.5 μg ml^−1^; D1306, Invitrogen) and F-actin using Alexa Fluor 647–phalloidin (1:100 dilution; A22287, Invitrogen), which were included in the secondary antibody incubations. Coverslips were mounted using ProLong Diamond antifade reagent (P36965, Invitrogen).

Images were acquired using a Zeiss 880 laser scanning microscope with Airyscan equipped with a Plan-Apochromat 63×/1.4 oil DIC M27 objective, or a Zeiss 710 confocal microscope equipped with Ec Plan Neofluar 40×/1.30 and Plan-apochromat 63×/1.40 oil objectives. All images were processed using Fiji software (ImageJ v2.0.0).

Focal adhesion analysis shown in Fig. 3B,C was performed using the Fiji software (ImageJ v2.0.0) and the Analyze Particles function, applying a minimum size threshold of 0.25 μm^2^. Cell shape analysis shown in Fig. S4A was performed by manually drawing around the cell perimeter, based on F-actin staining, and measuring the area and the shape descriptors using Fiji software (ImageJ v2.0.0). Cell shape analysis shown in Fig. S4C was performed using Cell Profiler software (v3.0.0; CellProfiler), applying a mask for cell area based on F-actin staining and a mask for nuclei. YAP nuclear to cytosolic ratios shown in Fig. S1B were calculated using the Fiji software (ImageJ v2.0.0) to quantify mean YAP fluorescence intensity on similar rectangular areas over and adjacent to the nucleus.

### Focal adhesion turnover

PDAC-A cells were transiently transfected with 8 µg paxillin–pEGFP plasmid using the Amaxa Cell Line Nucleofector kit and program P-031. The cells were plated onto 35-mm glass-bottom MatTek dishes pre-coated with either fibronectin, collagen VI or a 50:50 mix of the two. Short movies of 1 frame per min for 30 min were obtained using a Zeiss 880 confocal microscope with Airyscan using a Plan-Apochromat 63×/1.4 oil DIC objective lens and a 488 nm laser at 37°C and 5% CO_2_. Raw images were acquired, and Airyscan processing was performed using Zen Black version 2.3 SP1. Time-lapse movies were processed using Fiji software 1.53q. Image sequences were stabilised using the Fiji plugin Image Stabilizer, and a Gaussian blur (2.0) was applied to the image to highlight the focal adhesions. The tiff files were submitted to the Focal Adhesion Analysis Server (FAAS; http://faas.bme.unc.edu/) (Berginski and Gomez, 2013). A detection threshold of 3.5 was maintained across all image sets, and positive structures of 15 pixels^2^ that lasted for at least five consecutive frames were quantified as being a focal adhesion for the dynamic calculations. Assembly and disassembly rates were calculated using a previously described method (Webb et al., 2004) with modifications described by Berginski et al. (2011). FAAS also calculates dynamic properties such as focal adhesion longevity (lifetime). Data presented are from four independent experiments where *n*=30 for fibronectin, *n*=35 for collagen VI and *n*=33 for the fibronectin and collagen VI mix coatings.

### Immunohistochemistry

All Haematoxylin and Eosin (H&E), immunohistochemistry (IHC) and Picro Sirius red (PSR) staining was performed on 4 µm formalin-fixed paraffin-embedded (FFPE) sections that had previously been oven baked at 60°C for 2 h.

Sections were stained using the following antibodies on an Agilent Autostainer Link 48: anti-αSMA (A2547, Sigma-Aldrich), anti-collagen VI (ab182744, Abcam), anti-fibronectin (A0245, Agilent), anti-Ki-67 (RM-9106, monoclonal clone SP6, Lab Vision) and anti-p53 CM-5 (NCL-L-p53CM5p, Leica). FFPE sections were loaded into an Agilent pre-treatment module to be dewaxed and undergo heat-induced epitope retrieval (HIER) either using low or high pH target retrieval solution (TRS) (K8005, K8004, Agilent). Sections for αSMA staining underwent antigen retrieval using low pH TRS. Sections for collagen VI, fibronectin and p53 (also known as TP53) staining underwent antigen retrieval using high pH TRS. All sections were heated to 97°C for 20 min in the appropriate TRS. After HIER, all sections were rinsed in flex wash buffer (K8007, Agilent) prior to being loaded onto the autostainer. The sections then underwent peroxidase blocking (S2023, Agilent) for 5 min, before being washed with flex wash buffer. Sections for αSMA were blocked using mouse-on-mouse kit (MKB2213-1, Vector Lab) before primary antibody application for 35 min at a previously optimised dilution (αSMA, 1:25,000; collagen VI, 1:1000; fibronectin, 1:750; p53, 1:250). The sections were then washed with flex wash buffer before application of appropriate secondary antibody for 30 min. Sections for αSMA had mouse EnVision reagent (K4001, Agilent) applied for 30 min, and collagen VI, fibronectin, and p53 sections had rabbit EnVision reagent (K4003, Agilent) applied for 30 min. Sections were rinsed with flex wash buffer before applying Liquid DAB (K3468, Agilent) for 10 min. The sections were then washed in water and counterstained using Haematoxylin Z (RBA-4201-001 CellPath).

Sections were stained using the following antibodies on a Leica Bond Rx autostainer: anti-F4/80 (ab6640, Abcam) and anti-Ly6G (BE0075-1, BioXcell). All FFPE sections underwent on-board dewaxing (AR9222, Leica) and antigen retrieval using appropriate retrieval solution. Sections for F4/80 staining were retrieved using enzyme 1 solution (AR9551, Leica) for 10 min at 37°C. Sections for Ly6G underwent antigen retrieval using ER2 solution (AR9640, Leica) for 20 min at 95°C. Sections were rinsed with Leica wash buffer (AR9590, Leica) before peroxidase block was performed using an Intense R kit (DS9263, Leica) for 5 min. Sections were rinsed with wash buffer before all sections had the blocking solution applied from the Rat ImmPRESS kit (MP7444-15, Vector Labs) for 20 min. Sections were rinsed with wash buffer and then primary antibody was applied at an optimal dilution (F4/80, 1:100; Ly6G, 1:60,000). The sections were rinsed with wash buffer and had rat ImmPRESS secondary antibody (Vector Laboratories, MP-7404-50) applied for 30 min. The sections were rinsed with wash buffer and visualised using DAB in the Intense R kit.

H&E staining was performed on a Leica autostainer (ST5020). Sections were dewaxed, taken through graded alcohols and then stained with Haematoxylin Z for 13 mins. Sections were washed in water, differentiated in 1% acid alcohol and washed, and the nuclei were blued in Scott’s tap water substitute (in-house). After washing sections were placed in Putt's Eosin (in-house) for 3 min.

Staining for PSR was performed manually on FFPE sections that were dewaxed and rehydrated through xylene and a graded ethanol series before washing in water. Rehydrated slides were stained for 2 h in PSR staining solution [equal volumes of 0.1% Direct Red 80 (Sigma Aldrich) and 0.1% Fast Green (Raymond A Lamb), both in distilled water] combined in a 1:9 dilution with aqueous picric acid solution (VWR).

To complete H&E, IHC and PSR staining, sections were rinsed in tap water, dehydrated through a graded ethanol series and placed in xylene. The stained sections were mounted in xylene using DPX mountant (SEA-1300-00A, CellPath). Slides were scanned using a Leica Aperio AT2 slide scanner at 20× magnification, and all analyses were performed with HALO software (Indica labs).

### Random migration assay

Cells were plated at 20,000 cells per cm^2^ onto fibronectin-coated dishes and imaged for 16 h using a Nikon TE2000 microscope with a Plan Fluor 10×/0.30 objective and equipped with a heated CO_2_ chamber. Images were analysed with Fiji software (ImageJ v2.0.0).

### Inverted invasion assay

100 μl of 50% Matrigel in PBS solution was allowed to polymerise per Transwell for 30 min at 37°C. Cells were trypsinised, resuspended in medium and counted before 4×10^4^ cells were plated on the underside of the Transwell filter. Transwells were then placed inside 24-well plates so that the cell suspension droplets were in contact with the base of the 24-well plate, and then incubated for 2 h at 37°C allowing cell attachment to the bottom side of the filter. Transwells were then washed three times with 1 ml of serum-free medium. Chemotactic gradients were created by filling the upper chambers with medium containing 10% FBS, and the bottom chambers were kept in FBS-free medium. Cells were allowed to invade the Matrigel plug for 3.5 days and were stained for 60 min at 37°C with 4 μM of Calcein AM. Serial optical sections at 15-μm intervals were obtained using an Olympus FV1000 confocal microscope equipped with a UplanSApo 20×/0.74 objective. Images were analysed using Fiji software (ImageJ v2.0.0).

### Wound invasion assay

96-well Incucyte Imagelock plates were coated for 16 h with Matrigel (100 μg ml^−1^) at 37°C. PDAC cell monolayers were generated by plating 7×10^5^ cells per ml for 4 h. Monolayers were wounded using a 96-pin WoundMaker (Essen Bioscience) and embedded in Matrigel (2.15 mg ml^−1^, diluted in DMEM containing 10% FBS). Following 60 min incubation at 37°C, fully supplemented medium was added to each well and images were acquired at 60 min intervals using an IncuCyte Zoom system (Essen Bioscience). Wound recovery was analysed using the automated Incucyte Zoom software (Essen Bioscience) providing relative wound density over time.

### Circular invasion assay

Circular invasion assay was performed as described previously (Yu and Machesky, 2012) with slight modifications for the needs of the experiment. A culture insert (Ibidi) was positioned in the middle of a 6-well glass-bottom dish. The main well and inner wells of the insert were then coated with 10 μg/ml of collagen VI (ab7538, Abcam, lot Gr3210520-1) for 1 h at 37°C. 1×10^6^ PDAC-A cells were seeded around the insert and allowed to grow overnight. The insert and medium were removed, and 400 μl of Matrigel was added on top of the dish and left to set for 30 min at 37°C. Finally, 3 ml of complete medium was added to the dish prior to imaging. Images were taken every 10 min for 20 h on a Nikon TE2000-E inverted time-lapse microscope equipped with a motorised stage, a perfect focus system (PFS) and Metamorph software (Molecular Devices).

### Intraperitoneal and intrasplenic transplantation assays and KPC mice

A list of mice used for IHC staining is provided in Table S1. Mice were maintained in the Biological Services Unit of the Beatson Institute according to UK Home Office regulations and in compliance with EU Directive 2010/63 and the UK Animals (Scientific Procedures) Act 1986. All protocols and experiments were previously approved by the Animal Welfare and Ethical Review Body (AWERB) of the University of Glasgow and were accompanied by a UK Home Office project licence to J. Morton (70/8375, intrasplenic) and to L.M.M. (PE494BE48, intraperitoneal and KPC mice).

The IP transplantation assay of PDAC cells was performed as previously described (Juin et al., 2019). PDAC cells (1×10^6^) were resuspended in 100 μl PBS and introduced into each nude mouse (CD-1nu, females, 6-week old; Charles River Laboratories, Wilmington, MA) by IP injection. Tumour nodules were quantified in the mesenterium and the diaphragm following 14 days from injection.

The instrasplenic assay was performed as previously described (Papalazarou et al., 2020). Following anaesthesia and transverse incision exposing the spleen, PDAC cells (1×10^6^) in 100 μl PBS were injected in the spleen of nude mice (CD-1nu, females, 6-week old; Charles River Laboratories, Wilmington, MA). Surgical clips were typically removed 7 days after surgery, and mice were euthanised 21 days after inoculation. Liver tumour burden was calculated as the percentage of tumour-bearing liver lobes over the total number of liver lobes. In all experiments mice were randomly injected with control or KO cell lines.

### Statistics and reproducibility

All datasets were analysed and plotted using Prism 8 (v8.2.0; GraphPad Software) unless otherwise stated. Differences between groups were tested for normal distribution and analysed using the appropriate statistical test, as mentioned in each figure legend. Error bars represent the s.d., unless otherwise stated. All experiments were repeated at least three times independently unless otherwise stated in the figure legends. Sample sizes for *in vitro* studies were chosen to achieve at least *P*=0.05 according to our previous experience with these assays. The raw data from this study, including RNA sequencing data, are provided in Table S2.

## Supplementary Material

10.1242/joces.259978_sup1 Supplementary information
