# Supplementary material for: Collagen VI expression is negatively mechanosensitive in pancreatic cancer cells and supports the metastatic niche
Source: J Cell Sci. 2022 Dec 22;135(24):jcs259978. doi: 10.1242/jcs.259978 (PMC9845737; doi:10.1242/jcs.259978)
Supplement: Supplementary information [file joces-135-259978-s1.pdf]

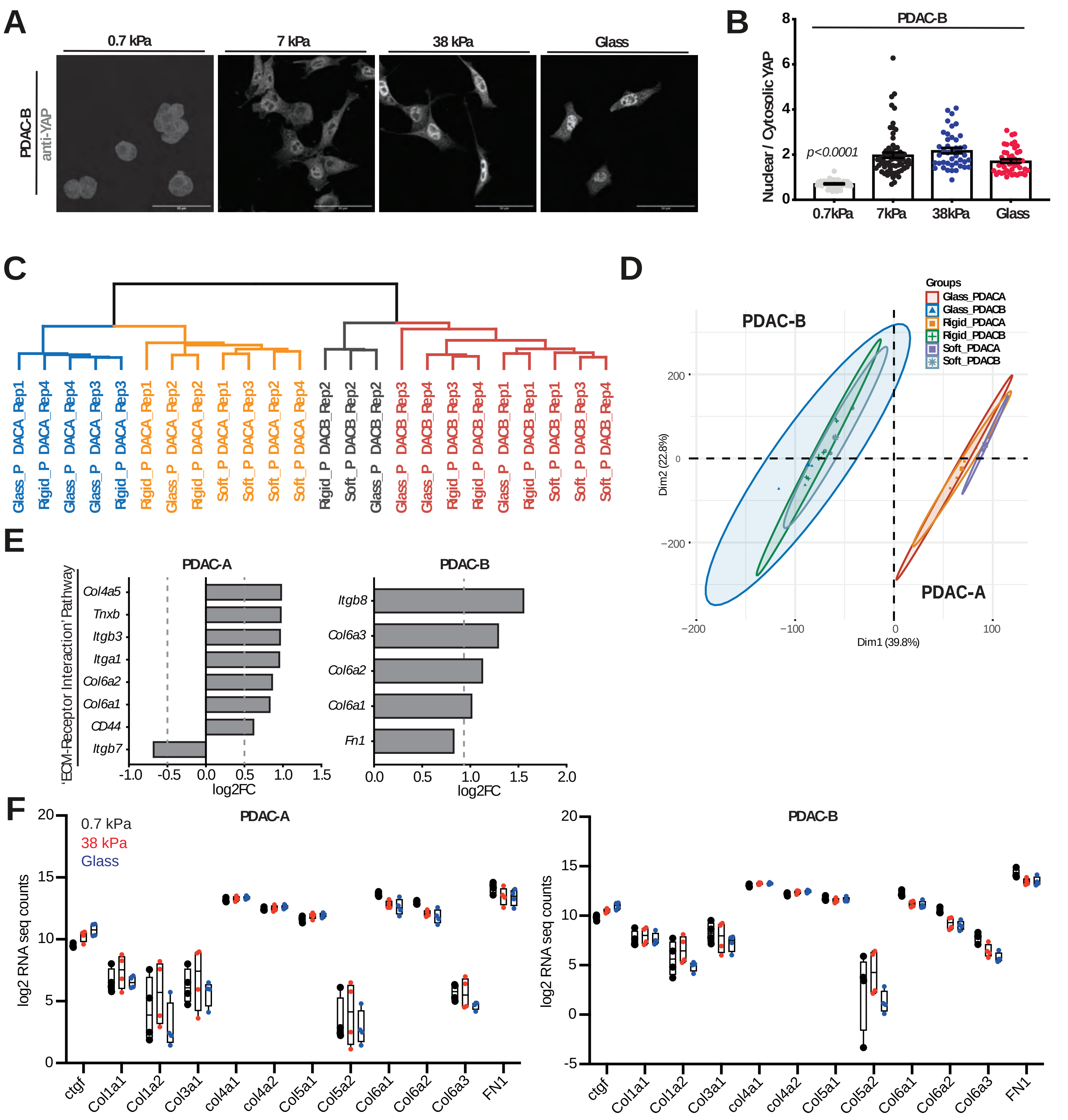
A
0.7 kPa
7 kPa
38 kPa
Glass
PDAC-B
anti-YAP
B
PDAC-B
Nuclear / Cytosolic YAP
p<0.0001
0.7kPa
7kPa
38kPa
Glass
C
D
PDAC-B
PDAC-A
Groups
Glass_PDACA
Glass_PDACB
Rigid_PDACA
Rigid_PDACB
Soft_PDACA
Soft_PDACB
Dim1 (39.8%)
Dim2 (22.8%)
E
PDAC-A
PDAC-B
'ECM-Receptor Interaction' Pathway
Col4a5
Tnxb
Itgb3
Itga1
Col6a2
Col6a1
CD44
Itgb7
Itgb8
Col6a3
Fn1
log2FC
F
PDAC-A
PDAC-B
0.7 kPa
38 kPa
Glass
log2 RNA seq counts
ctgf
Col1a1
Col1a2
Col3a1
col4a1
col4a2
Col5a1
Col5a2
Col6a1
Col6a2
Col6a3
FN1

Fig. S1. Low substrate stiffness alters the expression of matrisome-related genes in PDAC cells.

A: Immunofluorescence of PDAC-B cells cultured atop of 0.7 kPa, 7 kPa or 38 kPa fibronectin-coated polyacrylamide hydrogels for 24 hours, showing YAP1 (grey). Scale bars, 50 µm.

B: Quantification of 'nuclear YAP1'/ 'cytosolic YAP1' intensity ratio for cells in (A). Values are mean ± s.e.m. from n= 52 cells, 0.7kPa; n= 68, 7kPa; n= 42, 38kPa; n= 46, glass. Cells are from 3 independent experiments. Statistical significance was assessed by Kruskal-Wallis test with Dunn's multiple comparisons test.

C: Hierarchical clustering of RNA sequencing signatures from PDAC-A and PDAC-B cells cultured on fibronectin-coated 0.7 kPa, 38 PAAm hydrogels and glass coverslips for 24 hours.

D: PCA clustering of cells from **(C)**.

E: Bar plot displaying differentially expressed genes (p_{adj} < 0.05; log2 fold change >1) from the 'ECM-Receptor Interaction' KEGG pathway that were enriched in cells from **(C)**. Data is organised by log2 fold change.

F: log2 RNA seq counts of indicated genes genes of PDAC-A (left) and PDAC-B (right) cells cultured on 0.7 kPa, 38 kPa hydrogels and glass coverslips. Data is from n = 4 independent replicates per condition for each cell line.

Journal of Cell Science • Supplementary information

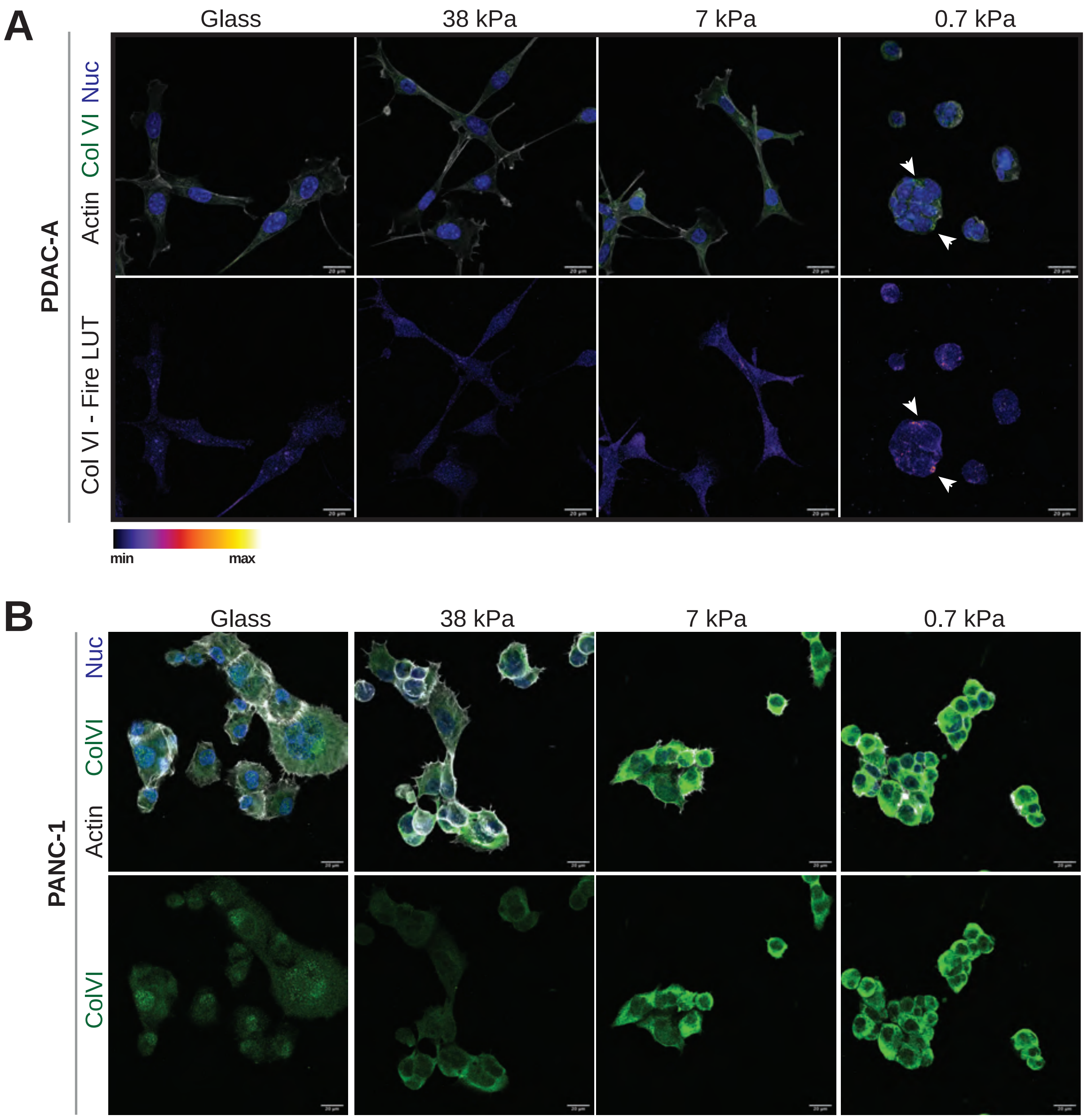

Journal of Cell Science • Supplementary information

Fig. S2. Collagen VI is upregulated in PDAC cells upon low substrate stiffness

A: Top; Immunofluorescence of PDAC-A cells cultured on glass coverslips, 0.7-, 7- and 38-kPa fibronectin-coated hydrogels, showing Collagen VI (green), Actin (grey) and nuclei (blue). Representative pictures from 3 independent experiments. Bottom; Individual Collagen VI channel (Fire LUT). Scale bars, 20µm. Arrowheads indicate Collagen VI enrichment.

B: Top; Immunofluorescence of PANC-1 cells cultured on glass coverslips, 0.7-, 7- and 38-kPa fibronectin-coated hydrogels, showing Collagen VI (green), Actin (grey) and nuclei (blue). Representative pictures from 3 independent experiments. Bottom; Individual Collagen VI channel (Green). Scale bars, 20µm.

Journal of Cell Science • Supplementary information

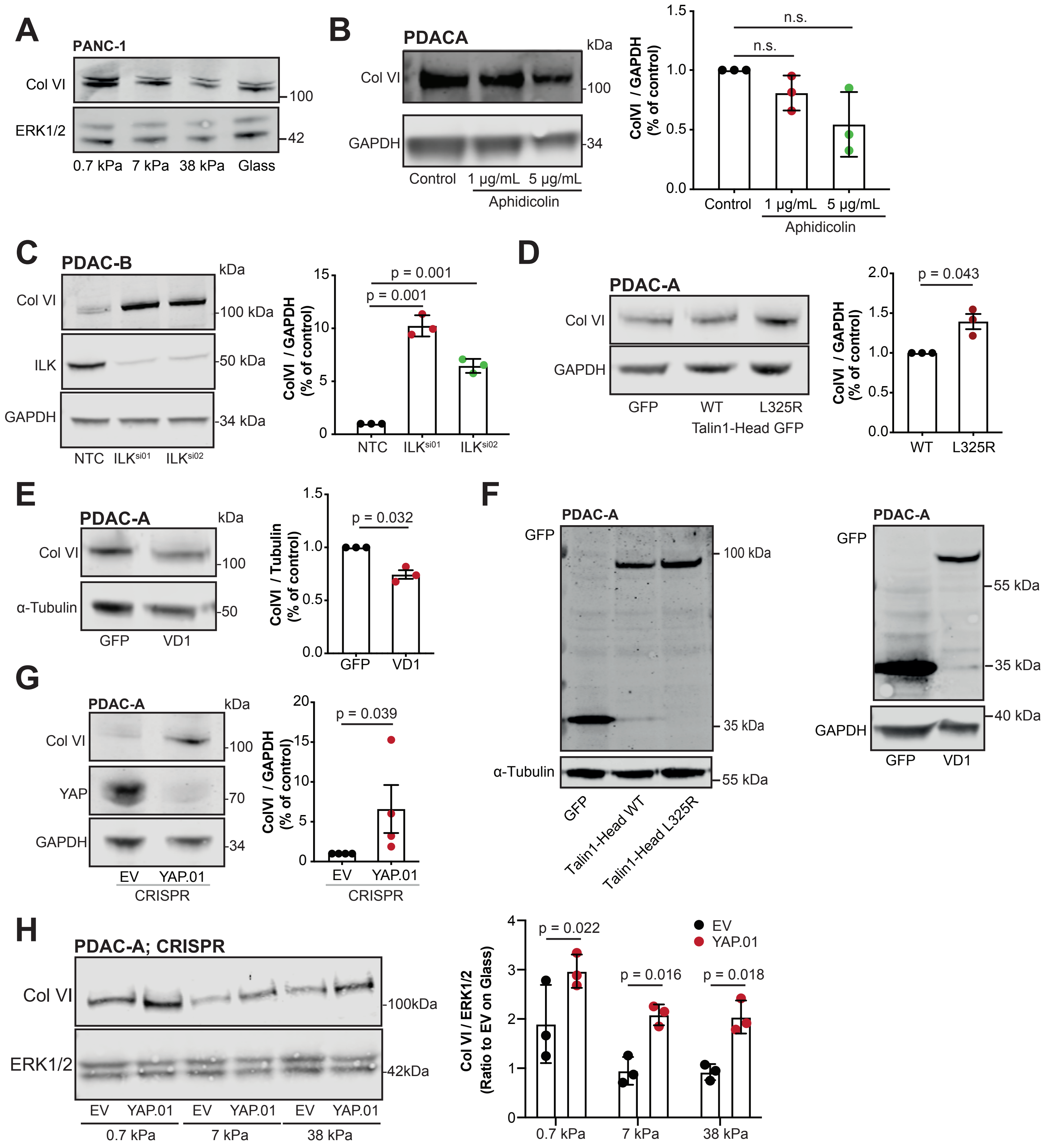

Journal of Cell Science • Supplementary information

Fig. S3. Loss of ECM adhesion and mechanosensing upregulates Collagen VI expression in PDAC cells.

A: Collagen VI protein expression in PANC-1 cells measured by immunoblotting for Collagen VI (Col VI) and ERK1/2 (loading control). Blots are representative of two independent experiments.

B: Left; PDAC-A cells were treated with 1 µg/mL and 5 µg/mL aphidicolin for 24 hours and immunoblotted for Collagen VI and GAPDH (loading control). Right; Densitometric quantification of protein expression. Values are mean ± s.d.

C: Left; Control (NTC) or ILK silenced (Ilk^{si01}, Ilk^{si02}) PDAC-B cells were immunoblotted for Collagen VI, ILK and α-Tubulin (loading control). Right; Densitometric quantification of protein expression. Values are mean ± s.d.

D: Left; PDAC-A cells expressing either GFP or GFP-tagged Talin 1-head domain (WT, control) or Talin-head L325R mutant (L325R) were immunoblotted for Collagen VI and GAPDH (loading control). Right; Densitometric quantification of protein expression. Values are mean ± s.d.

E: Left; PDAC-A cells expressing either GFP (control) or GFP-tagged Vinculin Domain 1 (VD1) were immunoblotted for Collagen VI and α-Tubulin (loading control). Right; Densitometric quantification of protein expression. Values are mean ± s.d.

F: Left; PDAC-A cells expressing expressing either GFP or GFP-tagged Talin 1-head domain (WT, control) or Talin-head L325R mutant (L325R) were immunoblotted for GFP and α-Tubulin (loading control). Right; PDAC-A cells expressing either GFP or GFP-tagged Vinculin Domain 1 (VD1) were immunoblotted for GFP and GAPDH (loading control).

G: Left; Control (EV) or YAP-depleted (YAP.01) PDAC-A cells were immunoblotted for Collagen VI, YAP and GAPDH (loading control). Pictures are representative of 4 independent experiments. Right; Densitometric quantification of ColVI protein expression. Values are mean ± s.d.

H: Left; Control (EV) or YAP-depleted (YAP.01) PDAC-A cells were cultured on fibronectin-coated 0.7- , 7- and 38-kPa hydrogels and were immunoblotted for Collagen VI and ERK1/2 (loading control). Right; Densitometric quantification of protein. Values are mean ± s.d. and representative from 3 independent experiments. Statistical significance was assessed by two-way ANOVA and p-values were corrected for multiple comparisons by Šídák's test.

All data in B-E are from 3 independent experiments. Statistical significance was assessed by two-tailed one-sample *t*-test on natural log-transformed values.

Journal of Cell Science • Supplementary information

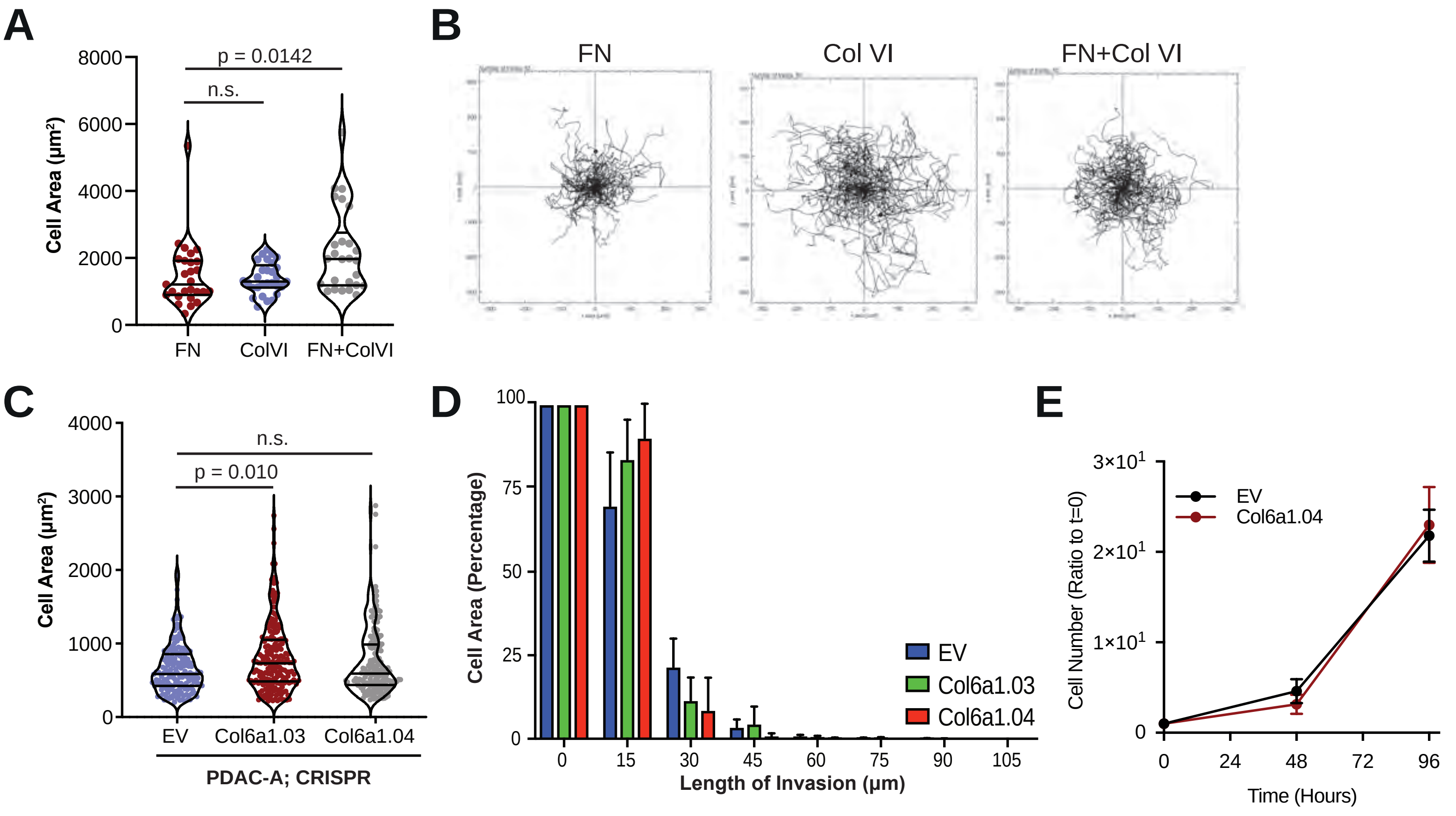

Fig. S4. Collagen VI ECM supports migratory behaviour of PDAC cells *in vitro* and loss of Col6a1 expression delays invasion through recombinant basement membrane ECM.

A: Cell area (µm^2) quantification of PDAC-A cells cultured on fibronectin (FN), collagen VI (ColVI) or fibronectin and collagen VI (FN+ColVI) glass coverslips. Values are from $n = 27$ FN cells; $n = 29$, ColVI; $n = 26$, Fn+ColVI cells. Cells are from three independent experiments. Statistical significance was assessed by Kruskal-Wallis with Dunn's multiple comparisons test.

B: Tracks (spider plots) of PDAC-A cells migrating on fibronectin (FN), collagen VI (ColVI) or fibronectin and collagen VI (FN+ColVI) glass coverslips for 16 hours.ho

C: Cell area (µm^2) quantification of Control (EV) or Collagen VI depleted (Col6a1.03 and Col6a1.04) mouse PDAC-A cells. Values are mean ± s.d. from n=170 EV, n=191 Col6a1.03 and n=183 Col6a1.04 cells from 3 independent experiments. Statistical significance was assessed by Kruskal-Wallis with Dunn's multiple comparisons test.

D: Quantification of invaded area of Control (EV) or Collagen VI depleted (Col6a1.03 and Col6a1.04) mouse PDAC-A cells invading through the inverted invasion assay setup. Intensity for each depth is reported as a percentage of intensity at 0 µm. Values are me an ± s.d. from 3 independent experiments.

E: Cell number (Ratio to t=0) over time of control (EV) or Collagen VI depleted (Col6a1.01-04) PDAC-B cells. Values are mean ± SD from n = 3 independent experiments.

Journal of Cell Science • Supplementary information

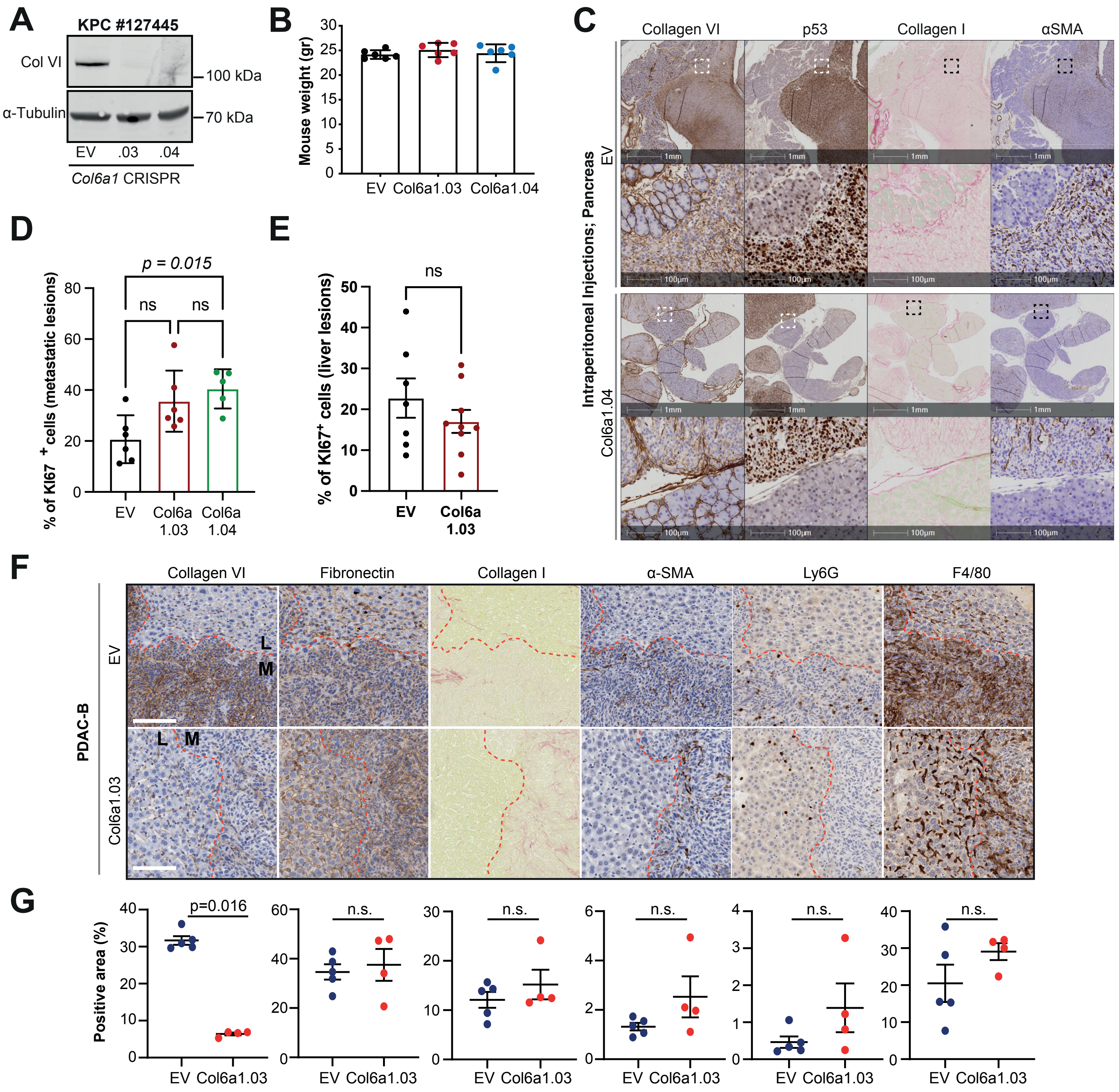

Journal of Cell Science • Supplementary information

Fig. S5. Collagen VI expression supports establishment of pancreatic metastasis in vivo.

A: Control (EV) or Collagen VI depleted (Col6a1.01-04) KPC cells were immunoblotted for Collagen VI and α-Tubulin (loading control).

B: Weight (gr) per mouse as indicated from intraperitoneal injection of control (EV) or Collagen VI depleted (Col6a1.03 and Col6a1.04) KPC cells after sacrifice. Values are mean ± SD from n = 6 EV, n = 6 Col6a1.03 and n = 6 Col6a1.04 mice.

C: Representative immunohistochemistry images showing Collagen VI, p53, Collagen I and α-SMA expression in tumors formed in the pancreas by intraperitoneal injection of control (EV) (top 2 panels) or Collagen VI depleted (bottom 2 panels) KPC cells. Scale bars, 1mm and 100μm.

D: Quantifications of $KI67^{+}$ cells in metastatic lesions of nude mice intraperitoneally injected with control (EV), or Collagen VI depleted KPC cells (Col6a1.03 and Col6a1.04). Mean ± s.e.m. from n = 6 EV, n = 6 Col6a1.03 and n = 5 Col6a1.04 mice. Statistical significance was assessed with Brown-Forsythe and Welch ANOVA test with Dunnett's multiple comparison test.

E: Quantifications of $KI67^{+}$ cells in metastatic lesions of nude mice intrasplenically injected with control (EV), or Collagen VI depleted PDAC-B (Col6a1.03). Mean ± s.e.m. from n = 7 EV and n = 9 Col6a1.03 mice. Statistical significance was assessed with two-tailed Welch's t-test.

F: Representative immunohistochemistry images showing Collagen VI, Fibronectin, Collagen I, α-SMA, Ly6G and F4/80 expression in liver metastatic nodules formed by intrasplenic injection of control (EV; top) or Collagen VI depleted (Col6a1.03; bottom) KPC cells. Red line denotes liver (L) and metastasis (M) boundary. Scale bars, 100μm.

G: Quantification of positively stained regions over tumor area (%) from D. Values are mean ± s.e.m. from n = 5 control (EV) and n = 4 Col6a1.03 mice. Statistical significance was assessed by Mann-Whitney test (Collagen VI) and unpaired t-test (Fibronectin, Collagen I, αSMA, Ly6G and F4/80).

Journal of Cell Science • Supplementary information

Table S1. Mice Information

Mouse ID	Strain	Genotype	Sex	Genetic Background	Age (days)	Application	Source
**KPC endpoint PDAC**							
FPZPR 103996	Pdx-1::Cre;LSL-Kras G12D;LSL-p53 R172H (KPC)	Pdx-1::Cre+;LSL-Kras G12D/+;LSL-p53 R172H/+ (KPC)	Male	C57BL/6J	118	IHC	CRUK Beatson Institute
BALF 162992	Pdx-1::Cre;LSL-Kras G12D;LSL-p53 R172H (KPC)	Pdx-1::Cre+;LSL-Kras G12D/+;LSL-p53 R172H/+ (KPC)	Male	C57BL/6J	123	IHC	CRUK Beatson Institute
BALF 192959	Pdx-1::Cre;LSL-Kras G12D;LSL-p53 R172H (KPC)	Pdx-1::Cre+;LSL-Kras G12D/+;LSL-p53 R172H/+ (KPC)	Male	C57BL/6J	233	IHC	CRUK Beatson Institute
BALF 192970	Pdx-1::Cre;LSL-Kras G12D;LSL-p53 R172H (KPC)	Pdx-1::Cre+;LSL-Kras G12D/+;LSL-p53 R172H/+ (KPC)	Female	C57BL/6J	114	IHC	CRUK Beatson Institute
BALA 27356	Pdx-1::Cre;LSL-Kras G12D;LSL-p53 R172H (KPC)	Pdx-1::Cre+;LSL-Kras G12D/+;LSL-p53 R172H/+ (KPC)	Male	C57BL/6J	140	IHC	CRUK Beatson Institute
BALA 32316	Pdx-1::Cre;LSL-Kras G12D;LSL-p53 R172H (KPC)	Pdx-1::Cre+;LSL-Kras G12D/+;LSL-p53 R172H/+ (KPC)	Female	C57BL/6J	152	IHC	CRUK Beatson Institute
BALA 32329	Pdx-1::Cre;LSL-Kras G12D;LSL-p53 R172H (KPC)	Pdx-1::Cre+;LSL-Kras G12D/+;LSL-p53 R172H/+ (KPC)	Female	C57BL/6J	94	IHC	CRUK Beatson Institute
BALF 6.1b	Pdx-1::Cre;LSL-Kras G12D;LSL-p53 R172H (KPC)	Pdx-1::Cre+;LSL-Kras G12D/+;LSL-p53 R172H/+ (KPC)	Male	C57BL/6J	170	IHC	CRUK Beatson Institute
BALF 6.1e	Pdx-1::Cre;LSL-Kras G12D;LSL-p53 R172H (KPC)	Pdx-1::Cre+;LSL-Kras G12D/+;LSL-p53 R172H/+ (KPC)	Female	C57BL/6J	166	IHC	CRUK Beatson Institute
BALA 22160	Pdx-1::Cre;LSL-Kras G12D;LSL-p53 R172H (KPC)	Pdx-1::Cre+;LSL-Kras G12D/+;LSL-p53 R172H/+ (KPC)	Female	C57BL/6J	99	IHC	CRUK Beatson Institute
BALA 24900	Pdx-1::Cre;LSL-Kras G12D;LSL-p53 R172H (KPC)	Pdx-1::Cre+;LSL-Kras G12D/+;LSL-p53 R172H/+ (KPC)	Female	C57BL/6J	94	IHC	CRUK Beatson Institute
BALA 32310	Pdx-1::Cre;LSL-Kras G12D;LSL-p53 R172H (KPC)	Pdx-1::Cre+;LSL-Kras G12D/+;LSL-p53 R172H/+ (KPC)	Male	C57BL/6J	139	IHC	CRUK Beatson Institute
**Normal**							
BALF 170396	Pdx-1::Cre;LSL-Kras G12D;LSL-p53 R172H (KPC)	Pdx-1::Cre+;LSL-Kras +/+;LSL-p53 +/+	Male	C57BL/6J	204	IHC	CRUK Beatson Institute
BALF 171667	Pdx-1::Cre;LSL-Kras G12D;LSL-p53 R172H (KPC)	Pdx-1::Cre+;LSL-Kras +/+;LSL-p53 +/+	Female	C57BL/6J	202	IHC	CRUK Beatson Institute
BALF 80410	Pdx-1::Cre;LSL-Kras G12D;LSL-p53 R172H (KPC)	Pdx-1::Cre+;LSL-Kras +/+;LSL-p53 +/+	Male	C57BL/6J	46	IHC	CRUK Beatson Institute
BALF 80418	Pdx-1::Cre;LSL-Kras G12D;LSL-p53 R172H (KPC)	Pdx-1::Cre+;LSL-Kras +/+;LSL-p53 +/+	Female	C57BL/6J	46	IHC	CRUK Beatson Institute
**KPC 10-week PanIN**							
BAJD 67.3c	Pdx-1::Cre;LSL-Kras G12D;LSL-p53 R172H (KPC)	Pdx-1::Cre+;LSL-Kras G12D/+;LSL-p53 R172H/+ (KPC)	Male	C57BL/6J	68	IHC	CRUK Beatson Institute
BAJD 91.1a	Pdx-1::Cre;LSL-Kras G12D;LSL-p53 R172H (KPC)	Pdx-1::Cre+;LSL-Kras G12D/+;LSL-p53 R172H/+ (KPC)	Female	C57BL/6J	69	IHC	CRUK Beatson Institute
BAJD 91.1d	Pdx-1::Cre;LSL-Kras G12D;LSL-p53 R172H (KPC)	Pdx-1::Cre+;LSL-Kras G12D/+;LSL-p53 R172H/+ (KPC)	Female	C57BL/6J	69	IHC	CRUK Beatson Institute
BAJD 91.1f	Pdx-1::Cre;LSL-Kras G12D;LSL-p53 R172H (KPC)	Pdx-1::Cre+;LSL-Kras G12D/+;LSL-p53 R172H/+ (KPC)	Female	C57BL/6J	69	IHC	CRUK Beatson Institute
BAJD 91.2b	Pdx-1::Cre;LSL-Kras G12D;LSL-p53 R172H (KPC)	Pdx-1::Cre+;LSL-Kras G12D/+;LSL-p53 R172H/+ (KPC)	Female	C57BL/6J	69	IHC	CRUK Beatson Institute
**KPC 15-week PanIN**							
BALF 11.2g	Pdx-1::Cre;LSL-Kras G12D;LSL-p53 R172H (KPC)	Pdx-1::Cre+;LSL-Kras G12D/+;LSL-p53 R172H/+ (KPC)	Female	C57BL/6J	104	IHC	CRUK Beatson Institute
BALF 18.2b	Pdx-1::Cre;LSL-Kras G12D;LSL-p53 R172H (KPC)	Pdx-1::Cre+;LSL-Kras G12D/+;LSL-p53 R172H/+ (KPC)	Female	C57BL/6J	103	IHC	CRUK Beatson Institute
BALF 18.2d	Pdx-1::Cre;LSL-Kras G12D;LSL-p53 R172H (KPC)	Pdx-1::Cre+;LSL-Kras G12D/+;LSL-p53 R172H/+ (KPC)	Female	C57BL/6J	103	IHC	CRUK Beatson Institute
BALF 18.2e	Pdx-1::Cre;LSL-Kras G12D;LSL-p53 R172H (KPC)	Pdx-1::Cre+;LSL-Kras G12D/+;LSL-p53 R172H/+ (KPC)	Female	C57BL/6J	103	IHC	CRUK Beatson Institute
BALF 11.2a	Pdx-1::Cre;LSL-Kras G12D;LSL-p53 R172H (KPC)	Pdx-1::Cre+;LSL-Kras G12D/+;LSL-p53 R172H/+ (KPC)	Male	C57BL/6J	104	IHC	CRUK Beatson Institute
**Intraperitoneal Injection Model - KPC cells**							
BVCD3.1a	Foxn1-nu:Hom (CD1-nu)	Foxn1-nu:Hom (CD1-nu); Injected KPC EV (WT) cells	Female	CD1	62	Intrasplenic	Charles River Laboratories
BVCD3.1b	Foxn1-nu:Hom (CD1-nu)	Foxn1-nu:Hom (CD1-nu); Injected KPC EV (WT) cells	Female	CD1	62	Intrasplenic	Charles River Laboratories
BVCD3.1c	Foxn1-nu:Hom (CD1-nu)	Foxn1-nu:Hom (CD1-nu); Injected KPC EV (WT) cells	Female	CD1	62	Intrasplenic	Charles River Laboratories
BVCD3.2a	Foxn1-nu:Hom (CD1-nu)	Foxn1-nu:Hom (CD1-nu); Injected KPC EV (WT) cells	Female	CD1	62	Intrasplenic	Charles River Laboratories
BVCD3.2b	Foxn1-nu:Hom (CD1-nu)	Foxn1-nu:Hom (CD1-nu); Injected KPC EV (WT) cells	Female	CD1	62	Intrasplenic	Charles River Laboratories
BVCD3.2c	Foxn1-nu:Hom (CD1-nu)	Foxn1-nu:Hom (CD1-nu); Injected KPC EV (WT) cells	Female	CD1	62	Intrasplenic	Charles River Laboratories
BVCD3.3a	Foxn1-nu:Hom (CD1-nu)	Foxn1-nu:Hom (CD1-nu); Injected KPC Col6a1.03 (KO) cells	Female	CD1	62	Intrasplenic	Charles River Laboratories
BVCD3.3b	Foxn1-nu:Hom (CD1-nu)	Foxn1-nu:Hom (CD1-nu); Injected KPC Col6a1.03 (KO) cells	Female	CD1	62	Intrasplenic	Charles River Laboratories
BVCD3.3c	Foxn1-nu:Hom (CD1-nu)	Foxn1-nu:Hom (CD1-nu); Injected KPC Col6a1.03 (KO) cells	Female	CD1	62	Intrasplenic	Charles River Laboratories
BVCD3.4a	Foxn1-nu:Hom (CD1-nu)	Foxn1-nu:Hom (CD1-nu); Injected KPC Col6a1.03 (KO) cells	Female	CD1	62	Intrasplenic	Charles River Laboratories
BVCF3.4b	Foxn1-nu:Hom (CD1-nu)	Foxn1-nu:Hom (CD1-nu); Injected KPC Col6a1.03 (KO) cells	Female	CD1	62	Intrasplenic	Charles River Laboratories
BVCD3.4c	Foxn1-nu:Hom (CD1-nu)	Foxn1-nu:Hom (CD1-nu); Injected KPC Col6a1.03 (KO) cells	Female	CD1	62	Intrasplenic	Charles River Laboratories
BVCD3.5a	Foxn1-nu:Hom (CD1-nu)	Foxn1-nu:Hom (CD1-nu); Injected KPC Col6a1.04 (KO) cells	Female	CD1	62	Intrasplenic	Charles River Laboratories
BVCD3.5b	Foxn1-nu:Hom (CD1-nu)	Foxn1-nu:Hom (CD1-nu); Injected KPC Col6a1.04 (KO) cells	Female	CD1	62	Intrasplenic	Charles River Laboratories
BVCD3.5c	Foxn1-nu:Hom (CD1-nu)	Foxn1-nu:Hom (CD1-nu); Injected KPC Col6a1.04 (KO) cells	Female	CD1	62	Intrasplenic	Charles River Laboratories
BVCD3.6a	Foxn1-nu:Hom (CD1-nu)	Foxn1-nu:Hom (CD1-nu); Injected KPC Col6a1.04 (KO) cells	Female	CD1	62	Intrasplenic	Charles River Laboratories
BVCD3.6b	Foxn1-nu:Hom (CD1-nu)	Foxn1-nu:Hom (CD1-nu); Injected KPC Col6a1.04 (KO) cells	Female	CD1	62	Intrasplenic	Charles River Laboratories
BVCD3.6c	Foxn1-nu:Hom (CD1-nu)	Foxn1-nu:Hom (CD1-nu); Injected KPC Col6a1.04 (KO) cells	Female	CD1	62	Intrasplenic	Charles River Laboratories
**Intrasplenic Transplantation Model - KPC cells**							
Cage 1 NM	Foxn1-nu:Hom (CD1-nu)	Foxn1-nu:Hom (CD1-nu); Injected KPC EV (WT) cells	Female	CD1	62	Intrasplenic	Charles River Laboratories
Cage 1 L	Foxn1-nu:Hom (CD1-nu)	Foxn1-nu:Hom (CD1-nu); Injected KPC EV (WT) cells	Female	CD1	62	Intrasplenic	Charles River Laboratories
Cage 1 R	Foxn1-nu:Hom (CD1-nu)	Foxn1-nu:Hom (CD1-nu); Injected KPC EV (WT) cells	Female	CD1	62	Intrasplenic	Charles River Laboratories
Cage 1 L+R	Foxn1-nu:Hom (CD1-nu)	Foxn1-nu:Hom (CD1-nu); Injected KPC EV (WT) cells	Female	CD1	62	Intrasplenic	Charles River Laboratories
Cage 2 NM	Foxn1-nu:Hom (CD1-nu)	Foxn1-nu:Hom (CD1-nu); Injected KPC EV (WT) cells	Female	CD1	62	Intrasplenic	Charles River Laboratories
Cage 2 L	Foxn1-nu:Hom (CD1-nu)	Foxn1-nu:Hom (CD1-nu); Injected KPC EV (WT) cells	Female	CD1	62	Intrasplenic	Charles River Laboratories
Cage 2 R	Foxn1-nu:Hom (CD1-nu)	Foxn1-nu:Hom (CD1-nu); Injected KPC Col6a1.04 (KO) cells	Female	CD1	62	Intrasplenic	Charles River Laboratories
Cage 2 L+R	Foxn1-nu:Hom (CD1-nu)	Foxn1-nu:Hom (CD1-nu); Injected KPC Col6a1.04 (KO) cells	Female	CD1	62	Intrasplenic	Charles River Laboratories
Cage 3 NM	Foxn1-nu:Hom (CD1-nu)	Foxn1-nu:Hom (CD1-nu); Injected KPC Col6a1.04 (KO) cells	Female	CD1	62	Intrasplenic	Charles River Laboratories
Cage 3 L	Foxn1-nu:Hom (CD1-nu)	Foxn1-nu:Hom (CD1-nu); Injected KPC Col6a1.04 (KO) cells	Female	CD1	62	Intrasplenic	Charles River Laboratories
Cage 3 R	Foxn1-nu:Hom (CD1-nu)	Foxn1-nu:Hom (CD1-nu); Injected KPC Col6a1.04 (KO) cells	Female	CD1	62	Intrasplenic	Charles River Laboratories
Cage 3 L+R	Foxn1-nu:Hom (CD1-nu)	Foxn1-nu:Hom (CD1-nu); Injected KPC Col6a1.04 (KO) cells	Female	CD1	62	Intrasplenic	Charles River Laboratories
**Intrasplenic Transplantation Model - PDAC B cells**							
Cage 1 NM	Foxn1-nu:Hom (CD1-nu)	Foxn1-nu:Hom (CD1-nu); Injected PDAC-B EV (WT) cells	Female	CD1	62	Intrasplenic	Charles River Laboratories
Cage 1 L	Foxn1-nu:Hom (CD1-nu)	Foxn1-nu:Hom (CD1-nu); Injected PDAC-B EV (WT) cells	Female	CD1	62	Intrasplenic	Charles River Laboratories
Cage 1 R	Foxn1-nu:Hom (CD1-nu)	Foxn1-nu:Hom (CD1-nu); Injected PDAC-B EV (WT) cells	Female	CD1	62	Intrasplenic	Charles River Laboratories
Cage 1 L+R	Foxn1-nu:Hom (CD1-nu)	Foxn1-nu:Hom (CD1-nu); Injected PDAC-B EV (WT) cells	Female	CD1	62	Intrasplenic	Charles River Laboratories
Cage 1 2L	Foxn1-nu:Hom (CD1-nu)	Foxn1-nu:Hom (CD1-nu); Injected PDAC-B EV (WT) cells	Female	CD1	62	Intrasplenic	Charles River Laboratories
Cage 2 NM	Foxn1-nu:Hom (CD1-nu)	Foxn1-nu:Hom (CD1-nu); Injected PDAC-B EV (WT) cells	Female	CD1	62	Intrasplenic	Charles River Laboratories
Cage 2 L	Foxn1-nu:Hom (CD1-nu)	Foxn1-nu:Hom (CD1-nu); Injected PDAC-B EV (WT) cells	Female	CD1	62	Intrasplenic	Charles River Laboratories
Cage 2 R	Foxn1-nu:Hom (CD1-nu)	Foxn1-nu:Hom (CD1-nu); Injected PDAC-B EV (WT) cells	Female	CD1	62	Intrasplenic	Charles River Laboratories
Cage 2 L+R	Foxn1-nu:Hom (CD1-nu)	Foxn1-nu:Hom (CD1-nu); Injected PDAC-B EV (WT) cells	Female	CD1	62	Intrasplenic	Charles River Laboratories
Cage 2 2L	Foxn1-nu:Hom (CD1-nu)	Foxn1-nu:Hom (CD1-nu); Injected PDAC-B EV (WT) cells	Female	CD1	62	Intrasplenic	Charles River Laboratories
Cage 3 NM	Foxn1-nu:Hom (CD1-nu)	Foxn1-nu:Hom (CD1-nu); Injected PDAC-B Col6a1.03 (KO) cells	Female	CD1	62	Intrasplenic	Charles River Laboratories
Cage 3 L	Foxn1-nu:Hom (CD1-nu)	Foxn1-nu:Hom (CD1-nu); Injected PDAC-B Col6a1.03 (KO) cells	Female	CD1	62	Intrasplenic	Charles River Laboratories
Cage 3 R	Foxn1-nu:Hom (CD1-nu)	Foxn1-nu:Hom (CD1-nu); Injected PDAC-B Col6a1.03 (KO) cells	Female	CD1	62	Intrasplenic	Charles River Laboratories
Cage 3 L+R	Foxn1-nu:Hom (CD1-nu)	Foxn1-nu:Hom (CD1-nu); Injected PDAC-B Col6a1.03 (KO) cells	Female	CD1	62	Intrasplenic	Charles River Laboratories
Cage 3 2L	Foxn1-nu:Hom (CD1-nu)	Foxn1-nu:Hom (CD1-nu); Injected PDAC-B Col6a1.03 (KO) cells	Female	CD1	62	Intrasplenic	Charles River Laboratories
Cage 4 NM	Foxn1-nu:Hom (CD1-nu)	Foxn1-nu:Hom (CD1-nu); Injected PDAC-B Col6a1.03 (KO) cells	Female	CD1	62	Intrasplenic	Charles River Laboratories
Cage 4 L	Foxn1-nu:Hom (CD1-nu)	Foxn1-nu:Hom (CD1-nu); Injected PDAC-B Col6a1.03 (KO) cells	Female	CD1	62	Intrasplenic	Charles River Laboratories
Cage 4 R	Foxn1-nu:Hom (CD1-nu)	Foxn1-nu:Hom (CD1-nu); Injected PDAC-B Col6a1.03 (KO) cells	Female	CD1	62	Intrasplenic	Charles River Laboratories
Cage 4 L+R	Foxn1-nu:Hom (CD1-nu)	Foxn1-nu:Hom (CD1-nu); Injected PDAC-B Col6a1.03 (KO) cells	Female	CD1	62	Intrasplenic	Charles River Laboratories
Cage 4 2L	Foxn1-nu:Hom (CD1-nu)	Foxn1-nu:Hom (CD1-nu); Injected PDAC-B Col6a1.03 (KO) cells	Female	CD1	62	Intrasplenic	Charles River Laboratories

Click here to download Table S1

Table S2. Raw data, including RNA sequencing data.

Click here to download Table S2

Journal of Cell Science • Supplementary information
