# Supplementary material for: Collagen VI expression is negatively mechanosensitive in pancreatic cancer cells and supports the metastatic niche
Source: J Cell Sci. 2022 Dec 22;135(24):jcs259978. doi: 10.1242/jcs.259978 (PMC9845737; doi:10.1242/jcs.259978)
Supplement: Supplementary file 1 [file joces-135-259978_review_history.pdf]

Collagen VI expression is negatively mechanosensitive in pancreatic cancer cells and supports the metastatic niche

Vasileios Papalazarou, James Drew, Amelie Juin, Heather J. Spence, Jamie Whitelaw, Colin Nixon, Manuel Salmeron-Sanchez and Laura M. Machesky
DOI: 10.1242/jcs.259978

Editor: Andrew Ewald

Review timeline

Original submission:	3 March 2022
Editorial decision:	19 April 2022
First revision received:	5 October 2022
Editorial decision:	14 November 2022
Second revision received:	16 November 2022
Accepted:	19 November 2022

Original submission

First decision letter

MS ID#: JOCES/2022/259978

MS TITLE: Collagen-VI expression is negatively mechanosensitive in pancreatic cancer cells and supports the metastatic niche

AUTHORS: Vasileios Papalazarou, James Drew, Amelie Juin, Heather J Spence, Colin Nixon, Manuel Salmeron-Sanchez, and Laura M. Machesky
ARTICLE TYPE: Research Article

We have now reached a decision on the above manuscript.

To see the reviewers' reports and a copy of this decision letter, please go to: https://submit-jcs.biologists.org and click on the 'Manuscripts with Decisions' queue in the Author Area. (Corresponding author only has access to reviews.)

As you will see, the reviewers share enthusiasm for the study but raise a number of substantial criticisms that prevent me from accepting the paper at this stage. They suggest, however, that a revised version might prove acceptable, if you can address their concerns. If you think that you can deal satisfactorily with the criticisms on revision, I would be pleased to see a revised manuscript. We would then return it to the reviewers. Please feel free to reach out if you have questions about any of the reviewer comments.

Please ensure that you clearly highlight all changes made in the revised manuscript. Please avoid using 'Tracked changes' in Word files as these are lost in PDF conversion.

I should be grateful if you would also provide a point-by-point response detailing how you have dealt with the points raised by the reviewers in the 'Response to Reviewers' box. Please attend to all of the reviewers' comments. If you do not agree with any of their criticisms or suggestions please explain clearly why this is so.

© 2022. Published by The Company of Biologists under the terms of the Creative Commons Attribution License (https://creativecommons.org/licenses/by/4.0/).

Reviewer 1

Advance summary and potential significance to field

In this manuscript, Papalazarou and colleagues investigate how pancreatic tumor cells respond to their matrix environment. They define a mechanoresponsive biochemical pathway by which tumor cells upregulate expression of Collagen VI in the presence of a mechanically soft microenvironment via altered integrin engagement and YAP. Using both in vitro and in vivo models the authors demonstrate that expression of ColVI by the tumor cells is required for invasion and metastasis, suggesting that the tumor cells may contribute to the increased desmoplasia that is a hallmark of PDAC and that likely contributes to its poor prognosis. This represents a significant and interesting area of cancer cell biology. Overall, the manuscript is very well prepared, the data are clear, and the rigor of the experimental approach and statistical analysis appears high.

There are just a few places where the conclusions would be better supported by a more specific experimental approach, as indicated below.

Comments for the author

1. I think the major open question is if the increased ColVI is actually secreted by the cancer cells? The overall conclusion suggests that tumor cell-derived ColVI is secreted and contributes to ECM stiffness, but the analysis focuses primarily on intracellular ColVI, and does not measure ColVI secreted/deposited. Can an ELISA, imaging, or other method be used to measure secreted ColVI in cultured cells on the different matrices? Similarly, in vivo images in Fig 5D focus on intracellular ColVI. It seems it would be technically challenging to determine the source of the secreted collagen in vivo, so the authors should soften claims about which cell type is contributing the ColVI in the tumors.

2. Related to this, is there a change in the tumor stiffness with ColVI knockout – can this be measured? If there is no change because the stromal cells can compensate and contribute ColVI as suggested, then does the ColVI knockout inhibit metastasis by a mechanism distinct from its deposition in the ECM?

3. While it is supportive to see a correlation between ColVI and reduced survival (Fig 4E), the interpretation may be somewhat complicated. First the authors may want to clarify that the PDAC data from the TCGA dataset includes stromal cells as well as PDAC tumor cells. The cellular composition of normal versus cancerous pancreas is completely different, so increased ColVI RNA in the PDAC samples likely represents the change in cell content (from primarily acinar cells to tumor and activated stromal cells). Second, if the tumor cells decrease their ColVI expression on stiff matrices, which is likely the case in PDAC tumors, it is interesting that keeping expression high correlates with worse prognosis. Would this suggest that those tumors are less stiff? Or that the tumor cells that do not downregulate ColVI are more aggressive? Or that the stromal cells may have their own distinct regulation of ColVI expression?

4. To really make the claim that the focal adhesions are less stably associated with the substrate, it seems measuring focal adhesion lifetime/turnover using live cell imaging would be preferable. Also, is aspect ratio the appropriate measurement of focal adhesions in Figure 3A-B? It appears total size is also markedly decreased.

5. What is the stiffness of the matrigel solution used in Figure 3? Can the 3D wound healing experiments in Fig 3H be done on a softer versus harder substrate? Perhaps the ColVI knockout cells are more defective in migration/invasion on a softer substrate, where it would normally be upregulated as an adaptation?

6. Is there any difference in survival or proliferation of the ColVI knockout cells (in vitro or in vivo) that might contribute to their difference in metastatic growth in vivo?

7. The authors might want to consider including a reference to the new paper by DiMartino (Nature Cancers 2022) related to tumor cell production of Collagen III to support dormancy in the

© 2022. Published by The Company of Biologists under the terms of the Creative Commons Attribution License (https://creativecommons.org/licenses/by/4.0/).

metastatic niche. While a different collagen in a different cancer type, it is a similar theme in tumor-cell derived collagen to support its growth at a distant site.

Reviewer 2

Advance summary and potential significance to field

PDAC remains to be a major challenge and it remains unclear how the massive desmoplasia and tissue stiffening, characteristic to these tumors, is functionally contributing to disease progression. In this carefully designed and well written study, the Machesky laboratory has investigated the mechanoresponses of PDAC cells. Using hydrogel cultures corresponding to a range of normal and disease relevant pancreatic tissue rigidities, they have investigated the effect of stiffness on gene expression. Their two distinct PDAC lines show very different gene expression profiles. However, one common nominator is soft-matrix supported mRNA levels of Col VI genes. The authors link Col VI expression to adhesion and YAP signaling and demonstrate Col VI induced invasion and migration in matrigel. They also show that cancer cell-derived Col VI supports invasive potential of PDAC cells in vivo.

Comments for the author

Overall, this a very interesting manuscript with novel and potentially clinically relevant observations. There are, however, a few controls missing. In addition, the authors should investigate whether the cancer cell derived Col VI is secreted and deposited to the ECM.

Points to address:

1) The quality of the westernblot data in Figure 2 could perhaps be improved.
These data are the foundation of the paper and, albeit the quantifications are supportive of rigidity-controlled expression of Col VI, these changes are not immediately obvious for the representative blots owing to uneven loading. Could the authors include western blot data also from the Panc-1 cells?

2) Figure S2, Fig. 3. Is Col VI secreted by the cells? If the COLVI is retained intracellularly (as shown in S2), how do the authors envision it contributing to altered cell ECM interactions and migration. Can the authors stain for COLVI in their inverted invasion or migration assays? Are the control cells laying down MG-decorating Col VI?

3) What is the link between integrin activity and ColVI expression? The authors have used several way to disrupt the adhesion mediated mechanotransduction and find that ILK silencing in cells on plastic inhibits COLVI expression. They have also investigated the outcome of vinculin head (VD1) and talin-head fragments expression in cells cultured on plastic. The data are a little hard to interpret. Figure S3C, D. Could the authors perhaps compare the effects of WT talin head and the L325R mutant to cells transfected with a control plasmid. Now this control seems to be missing and the data are difficult to interpret. The authors should also demonstrate the expression levels of the Talin-head and VD1 constructs in their blots. WT-talin head should presumably activate integrins. Is the L325R mutant increasing Col VI or rather the wt talin head decreasing Col VI levels? The authors should describe accurately their VD1 data and mention that it significantly downregulates Col VI. There are some reports indicating VD1 activation of integrins (https://doi.org/10.1002/jcb.24772 and PMID: 20728432), albeit other studies have used this construct as a dominant-negative in cells. Thus, these data might suggest that increased integrin activity would be linked to decreased ColVI expression even on plastic. This would be in line with the authors conclusion of soft matrix upregulating COL VI. Would be interesting if they could explore this a bit more by testing the outcome of talin-head expression in cells on 0.7 kDa.

4) Figure 4. The changes in ColVI expression between normal and the PDAC progression samples are very clear indeed. It would be important, however, for the authors to demonstrate that their COL VI antibody is specific. Perhaps this could be done using FFPE samples prepared from the ColVI KO and control PDAC cells. For the conclusions of the paper, it would be pertinent to distinguish increased COL VI secretion from the overall desmoplasia which is a hallmark of PDAC progression.

© 2022. Published by The Company of Biologists under the terms of the Creative Commons Attribution License (https://creativecommons.org/licenses/by/4.0/).

Establishing the antibody specificity would also support the authors conclusion that the remaining Col VI signal in the Col VI KO tumors (Figure 5) would be derived from stromal cells.

Reviewer 3

Advance summary and potential significance to field

The authors describe for pancreatic cancers a pro-tumorigenic role for Col6, a fibrillar collagen associated with fibrosis (i.e. Col1,3,5, etc.) in other studies, and the present authors also present in vitro studies that shed light on pro-tumorigenic mechanism and suggest Col6 is "negatively mechanosensitive".

The latter is a potentially interesting advance.

Comments for the author

The authors describe for pancreatic cancers a pro-tumorigenic role for Col6, a fibrillar collagen associated with fibrosis (i.e. Col1,3,5, etc.) in other studies, and the present authors also present in vitro studies that shed light on pro-tumorigenic mechanism and suggest Col6 is "negatively mechanosensitive".
The main concerns with the in vitro studies are the mechanism and specificity of Col6 expression.

1. In terms of pathways sketched in Fig.2E and alluded to in the abstract, the idea that fibrillar collagen Col6 increases 'DUE to low YAP activity' is surprising and needs more evidence or qualification. I see in Fig.1D that the YAP target gene CTGF is low as expected on soft gels (but not 1C, curiously), but the effect on Col6 is puzzling. This is because the literature generally indicates CTGF is part of the fibrillar ECM and fibrosis pathway, and some studies indicates that includes COL6: "Collagen VI as a driver and disease biomarker in human fibrosis" [DOI: 10.1111/febs.16039]. Indeed, Fig.1D (and 1E) also shows higher fibronectin, which often precedes fibrillar collagen deposition. Although soft matrix and siYAP show similar effects, both generally cause cell rounding (per Fig.S1A's 0.7kPa) and rounding can also trigger gene expression separate from direct regulation by YAP. At least as a thought experiment, if cells were confined on patterns that limited cell spreading would Col6 be high and would it also increase/decrease with YAP knockdown/overexpression? What would happen to Fn1 and also Col1,3,5? Put another way, do the authors believe YAP is not only a transcriptional (co)activator of CTGF but also a direct transcriptional (co)repressor of Fn1 and Col6 (and maybe Col1,3,5)? Could the latter be indirect effects? For example the cells somehow sense a deficit in fibrillar ECM when they are rounded; alternatively, cell cycle is protracted on soft matrix (as is typical), and this leads to retention/accumulation of expressed genes that define lineage.

2. What do Fig.4D,E look like for other fibrosis genes (CTGF, Col1,3,5, etc.)?

First revision

Author response to reviewers' comments

Dear Andrew and Reviewers,
We thank you for your helpful and constructive review of our manuscript. We have attempted to respond to all of the points of the reviewers and to perform new experiments where needed. I would like to point out that we added Dr Jamie Whitelaw as a new co-author, because both James Drew and Vassilis Papalazarou have now left my lab. We hope that you will agree that we have improved our study and answered the reviewers' points satisfactorily.

© 2022. Published by The Company of Biologists under the terms of the Creative Commons Attribution License (https://creativecommons.org/licenses/by/4.0/).

Reviewer 1

Advance Summary and Potential Significance to Field:

In this manuscript, Papalazarou and colleagues investigate how pancreatic tumor cells respond to their matrix environment. They define a mechanoresponsive biochemical pathway by which tumor cells upregulate expression of Collagen VI in the presence of a mechanically soft microenvironment via altered integrin engagement and YAP. Using both in vitro and in vivo models the authors demonstrate that expression of ColVI by the tumor cells is required for invasion and metastasis, suggesting that the tumor cells may contribute to the increased desmoplasia that is a hallmark of PDAC and that likely contributes to its poor prognosis. This represents a significant and interesting area of cancer cell biology. Overall, the manuscript is very well prepared, the data are clear, and the rigor of the experimental approach and statistical analysis appears high. There are just a few places where the conclusions would be better supported by a more specific experimental approach, as indicated below.

We thank the reviewer for their constructive remarks on the manuscript.

Comments for the Author:

1. I think the major open question is if the increased ColVI is actually secreted by the cancer cells? The overall conclusion suggests that tumor cell-derived ColVI is secreted and contributes to ECM stiffness, but the analysis focuses primarily on intracellular ColVI, and does not measure ColVI secreted/deposited. Can an ELISA, imaging, or other method be used to measure secreted ColVI in cultured cells on the different matrices? Similarly, in vivo images in Fig 5D focus on intracellular ColVI. It seems it would be technically challenging to determine the source of the secreted collagen in vivo, so the authors should soften claims about which cell type is contributing the ColVI in the tumors.

We agree with the reviewer that determining ColVI secretion is a key point so we have performed additional experiments to address these comments. Regarding cellular collagen VI secretion, we cultured cells on fibronectin and concanavalin A coated plates and we measured collagen VI presence in the medium and the cellular lysates. As expected, intracellular collagen VI was increased upon loss of downstream integrin signalling in concanavalin coated surfaces (Figure 2G). This was followed by increased collagen VI presence in the medium confirming that collagen VI upregulation is followed by secretion in these cells (new panels in Figure 2G).

Regarding the in vivo stainings, it is indeed difficult in heavily cellularized tumours as the metastases in Fig 6D (ex Fig 5D) to delineate which cells exactly deposit this protein. In the metastases established by collagen VI KO cells as the ones in the middle and bottom panels in the figure, the cancer cells completely lack collagen VI expression, so the collagen VI detected in these does not originate from cancer cells and most likely is deposited by fibroblasts or other stromal cells. Moreover, extracellular collagen VI is more fibrillar when compared to the staining in the metastases from the control, Collagen VI-expressing cells, and looks to follow a similar pattern as aSMA staining. However, we agree that we cannot absolutely determine the origin of this collagen, so we have added a statement to clarify this.

While we cannot determine the origin of this surrounding ColVI in Col6a1 KO tumours, the tumour associated fibroblasts are the most likely source.

2. Related to this, is there a change in the tumor stiffness with ColVI knockout – can this be measured? If there is no change because the stromal cells can compensate and contribute ColVI as suggested, then does the ColVI knockout inhibit metastasis by a mechanism distinct from its deposition in the ECM?

This is a very interesting point; while collagen I, hyaluronan acid and fibronectin are considered to be the main drivers of ECM mechanical properties, absence of collagen VI may contribute to a different crosslinking and organisation of extracellular matrix. While

© 2022. Published by The Company of Biologists under the terms of the Creative Commons Attribution License (https://creativecommons.org/licenses/by/4.0/).

this is technically difficult to measure convincingly, we wouldn't expect to have major differences as we detected collagen VI presence even in metastatic lesions formed by collagen VI knockout cells. We reckon that collagen VI KO cells have a disadvantage in invading and seeding, but this can be partially compensated by collagen VI deposition by stromal cells. We have discussed this point in the discussion section (see lines 624-628).

3. While it is supportive to see a correlation between ColVI and reduced survival (Fig 4E), the interpretation may be somewhat complicated. First the authors may want to clarify that the PDAC data from the TCGA dataset includes stromal cells as well as PDAC tumor cells. The cellular composition of normal versus cancerous pancreas is completely different, so increased ColVI RNA in the PDAC samples likely represents the change in cell content (from primarily acinar cells to tumor and activated stromal cells).

We agree with the reviewer that TCGA expression data are derived from a mixture of cell types. Indeed, Collagen VI in primary mouse PDAC (Fig. 5B) appears stromal and its deposition is highly possible to be driven by cancer associated fibroblasts and other stromal cells. We added text to clarify this (lines 632-644).

Second, if the tumor cells decrease their ColVI expression on stiff matrices, which is likely the case in PDAC tumors, it is interesting that keeping expression high correlates with worse prognosis. Would this suggest that those tumors are less stiff? Or that the tumor cells that do not downregulate ColVI are more aggressive? Or that the stromal cells may have their own distinct regulation of ColVI expression?

These are interesting points and generally the cell-type of origin of ECM components emerges as an important question in cancer biology. We expanded the discussion at lines 624-628 to reflect this. Stiffness in PDAC tissue can be heterogeneous across the tumour, with areas of high/low stiffness. Indeed, it is intriguing to think that escaper cancer cells from areas with low stiffness (high Collagen-VI) expression have an advantage in establishing metastases. This could be perhaps contradictory to current dogma suggesting that cancer cells from areas of increased stiffness are more invasive and therefore more metastatic, but unfortunately this would be hard to test experimentally in the lab.

4. To really make the claim that the focal adhesions are less stably associated with the substrate, it seems measuring focal adhesion lifetime/turnover using live cell imaging would be preferable. Also, is aspect ratio the appropriate measurement of focal adhesions in Figure 3A-B? It appears total size is also markedly decreased.

We updated Figure 3 with focal adhesion lifetime measurements suggesting less stable focal adhesions on substrates that are coated with Collagen VI. We also added a plot of the quantification of average focal adhesion area (Figure 3C). This has brought in Dr. Jamie Whitelaw as a co-author as he performed these experiments.

5. What is the stiffness of the matrigel solution used in Figure 3? Can the 3D wound healing experiments in Fig 3H be done on a softer versus harder substrate? Perhaps the ColVI knockout cells are more defective in migration/invasion on a softer substrate, where it would normally be upregulated as an adaptation?

AFM measurements from others have calculated the stiffness of Matrigel at around 450 Pa (PMID: 19481153). In our experiments we used a 50% dilution of Matrigel in media, so the gels would be softer that the estimated stiffness of Matrigel. Therefore in terms of stiffness it is comparable to soft polyacrylamide gels. However, Matrigel contains a diverse mixture of proteins and growth factors compared to the fibronectin-coating of polyacrylamide gels. This could offer other factors that allow cells to compensate for Collagen VI loss, but would be difficult to delineate experimentally. However, we performed an experiment on Matrigel plugs supplemented with extracellular Collagen VI protein, and upon this scenario Collagen-VI KO cells invaded similar to control, indicating that extracellular collagen VI presence facilitates invasion (Fig.4J-K).

© 2022. Published by The Company of Biologists under the terms of the Creative Commons Attribution License (https://creativecommons.org/licenses/by/4.0/).

6. Is there any difference in survival or proliferation of the ColVI knockout cells (in vitro or in vivo) that might contribute to their difference in metastatic growth in vivo?

> We added Figure S4E showing that ColVI KO cells proliferate at similar rates as their controls in vitro. We also quantified proliferation of ColVI KO cells in vivo by ki67 staining in metastatic lesions formed either after intraperitoneal injections (Fig S5D) or after intrasplenic injections (Fig S5E). In both situations, ColVI KO didn't show defects in their proliferation that could explain a difference in their metastatic growth.

7. The authors might want to consider including a reference to the new paper by DiMartino (Nature Cancers 2022) related to tumor cell production of Collagen III to support dormancy in the metastatic niche. While a different collagen in a different cancer type, it is a similar theme in tumor-cell derived collagen to support its growth at a distant site.

> We thank the reviewer for suggesting this interesting study. Reference and comment added on lines 641, page 18.

Reviewer 2

Advance Summary and Potential Significance to Field:

PDAC remains to be a major challenge and it remains unclear how the massive desmoplasia and tissue stiffening, characteristic to these tumors, is functionally contributing to disease progression. In this carefully designed and well written study, the Machesky laboratory has investigated the mechanoresponses of PDAC cells. Using hydrogel cultures corresponding to a range of normal and disease relevant pancreatic tissue rigidities, they have investigated the effect of stiffness on gene expression. Their two distinct PDAC lines show very different gene expression profiles. However, one common nominator is soft-matrix supported mRNA levels of Col VI genes. The authors link Col VI expression to adhesion and YAP signaling and demonstrate Col VI induced invasion and migration in matrigel. They also show that cancer cell-derived Col VI supports invasive potential of PDAC cells in vivo.

Comments for the Author:

Overall, this a very interesting manuscript with novel and potentially clinically relevant observations. There are, however, a few controls missing. In addition, the authors should investigate whether the cancer cell derived Col VI is secreted and deposited to the ECM.

Points to address:

1) The quality of the western blot data in Figure 2 could perhaps be improved. These data are the foundation of the paper and, albeit the quantifications are supportive of rigidity-controlled expression of Col VI, these changes are not immediately obvious for the representative blots owing to uneven loading. Could the authors include western blot data also from the Panc-1 cells?

> It has been technically challenging to achieve equal loading from protein extracted by polyacrylamide gels especially of low stiffness. However even with a bit less protein on the 0.7 kPa conditions when compared to the other conditions, it is clear that cells produce more Collagen VI (still higher expression that the other conditions).
>
> Western blot data from Panc-1 cells has been added on Fig S3A.

2) Figure S2, Fig. 3. Is Col VI secreted by the cells? If the COLVI is retained intracellularly (as shown in S2), how do the authors envision it contributing to altered cell ECM interactions and migration. Can the authors stain for COLVI in their inverted invasion or migration assays? Are the control cells laying down MG-decorating Col VI?

> While collagen-VI does appear to accumulate within cells, the images in Fig S2 can just reflect increased Col VI expression (as suggested by RNAseq and WB data), which might be accompanied by secretion either onto the substratum or into the medium. To address this

© 2022. Published by The Company of Biologists under the terms of the Creative Commons Attribution License (https://creativecommons.org/licenses/by/4.0/).

point experimentally, we cultured cells on fibronectin (pro-integrin engagement substrate) and concanavalin A (anti- integrin engagement substrate) coated plates and we measured collagen VI presence in the media and the cellular lysates. As expected, intracellular collagen VI was increased upon loss of downstream integrin signalling in concanavalin coated surfaces. This was followed by increased collagen VI presence in the medium, confirming that collagen VI upregulation is accompanied by secretion in these cells (Fig. 2G).

Regarding the invasion assays, we performed new invasion experiments using mixed Matrigel+Col VI containing assays; indeed offering extracellular Collagen VI rescued the invasion defects showed by Collagen VI KO cells, suggesting that cancer cell invasion depends on extracellular Collagen VI (Fig.4J-K).

3) What is the link between integrin activity and ColVI expression?
The authors have used several ways to disrupt the adhesion mediated mechanotransduction and find that ILK silencing in cells on plastic inhibits COLVI expression. They have also investigated the outcome of vinculin head (VD1) and talin-head fragments expression in cells cultured on plastic. The data are a little hard to interpret.
Figure S3C, D. Could the authors perhaps compare the effects of WT talin head and the L325R mutant to cells transfected with a control plasmid. Now this control seems to be missing and the data are difficult to interpret.

To address this point, we have added in Fig S3D Collagen VI expression in cells expressing just GFP. However, we believe the best comparison to the L325R talin head mutant is the WT talin head.

The authors should also demonstrate the expression levels of the Talin-head and VD1 constructs in their blots.

We have now added panel S3F showing expression levels of Talin head and VD1 constructs.

WT-talin head should presumably activate integrins. Is the L325R mutant increasing Col VI or rather the wt talin head decreasing Col VI levels?

Based on Figure S3D the L325R mutant is increasing Col VI.

The authors should describe accurately their VD1 data and mention that it significantly downregulates Col VI.

We expanded our description of this data on lines 481-485.

There are some reports indicating VD1 activation of integrins (https://doi.org/10.1002/jcb.24772 and PMID: 20728432), albeit other studies have used this construct as a dominant-negative in cells. Thus, these data might suggest that increased integrin activity would be linked to decreased ColVI expression even on plastic. This would be in line with the authors conclusion of soft matrix upregulating COL VI. Would be interesting if they could explore this a bit more by testing the outcome of talin-head expression in cells on 0.7 kDa.

We briefly commented on this on lines 480-485. It is unlikely that talin-head expression per se would be enough to rescue the loss of mechanosensing on 0.7 kPa hydrogels and as both VP and JD have left from the lab, we have not been able to perform these experiments.

4) Figure 4. The changes in ColVI expression between normal and the PDAC progression samples are very clear indeed. It would be important, however, for the authors to demonstrate that their COL VI antibody is specific.

Please see below Figure demonstrating that the Collagen VI antibody we used (Abcam; ab182744) is specific to Collagen VI in our hands. Ovary is rich in ColVI, while in liver, this is confined to the peri-sinusoidal areas where hepatic stellate cells reside. Evidence is

© 2022. Published by The Company of Biologists under the terms of the Creative Commons Attribution License (https://creativecommons.org/licenses/by/4.0/).

also provided that this antibody recognises ColVI from the dramatic decrease in staining of our ColVI knockout tumours in Figure 6. Also, this antibody has been validated by the manufacturer using knockout studies and has been extensively used in many published peer-reviewed articles to demonstrate Collagen VI expression, including:

- 'Authentication of collagen VI antibodies' - DOI: 10.1186/s13104-017-2674-x
- 'Glycosaminoglycan Modification of Decorin Depends on MMP14 Activity and Regulates CollagenAssembly' - DOI: 10.3390/cells9122646
- 'Use of RNA‑sequencing to detect abnormal transcription of the collagen α‑2 (VI) chain gene that can lead to Bethlem myopathy' - DOI: 10.3892/ijmm.2021.4861
- 'Perfect chronic skeletal muscle regeneration in adult spiny mice, Acomys cahirinus' - DOI: 10.1038/s41598-018-27178-7
- 'BMP-dependent, injury-induced stem cell niche as a mechanism of heterotopic ossification' - DOI: 10.1186/s13287-018-1107-7

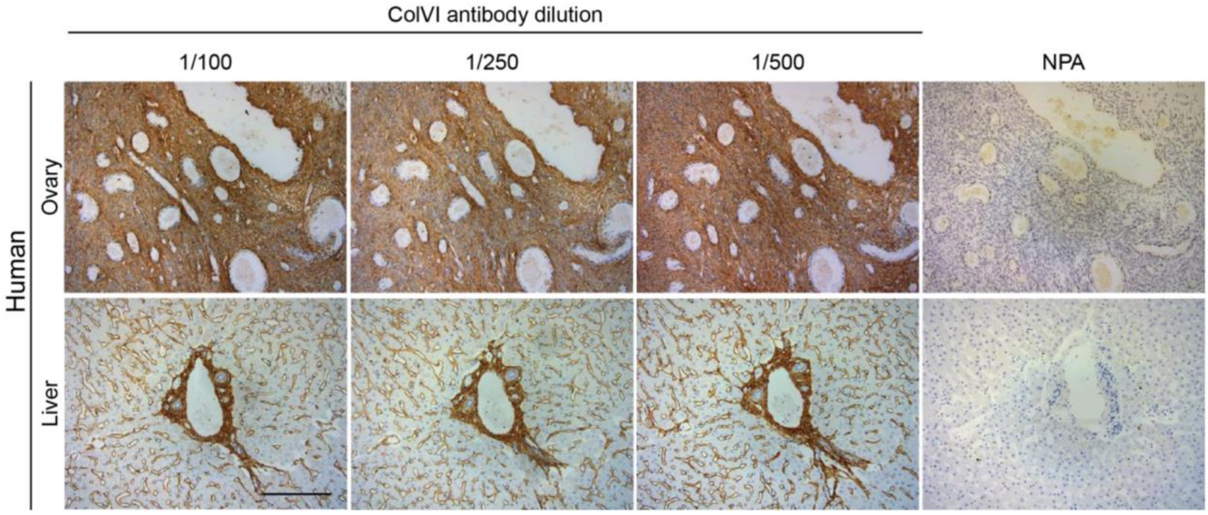

Figure 1 Serial sections of human liver and ovary stained with a serial dilution of the Collagen VI antibody used in this paper. NPA = no primary antibody. Top panel, the ovarian stroma presents a strong expression of collagen VI as previously shown in Pietila et al (Nature Communications 2021, co-evolution of matrisome and adaptative adhesion dynamics drives ovarian cancer chemoresistance). Bottom panel, Collagen VI is lining the sinusoids of the liver lobule and concentrates at the stroma of the portal tract consistent with the collagen VI stainings shown in Veidal et al, MMP Mediated Degradation of Type VI Collagen Is Highly Associated with Liver Fibrosis – Identification and Validation of a Novel Biochemical Marker Assay. Scale bar = 200um.

For the conclusions of the paper, it would be pertinent to distinguish increased COL VI secretion from the overall desmoplasia which is a hallmark of PDAC progression. Establishing the antibody specificity would also support the authors conclusion that the remaining Col VI signal in the Col VI KO tumors (Figure 5) would be derived from stromal cells.

We have added some discussion of this point on lines 624-628, p17.

Reviewer 3

Advance Summary and Potential Significance to Field:

The authors describe for pancreatic cancers a pro-tumorigenic role for Col6, a fibrillar collagen associated with fibrosis (i.e. Col1,3,5, etc.) in other studies, and the present authors also present in vitro studies that shed light on pro-tumorigenic mechanism and suggest Col6 is "negatively mechanosensitive". The latter is a potentially interesting advance.

© 2022. Published by The Company of Biologists under the terms of the Creative Commons Attribution License (https://creativecommons.org/licenses/by/4.0/).

Comments for the Author:

The authors describe for pancreatic cancers a pro-tumorigenic role for Col6, a fibrillar collagen associated with fibrosis (i.e. Col1,3,5, etc.) in other studies, and the present authors also present in vitro studies that shed light on pro-tumorigenic mechanism and suggest Col6 is "negatively mechanosensitive". The main concerns with the in vitro studies are the mechanism and specificity of Col6 expression.

Almost all ECM proteins, including collagens, fibronectin, hyaluronan acid are a part of a fibrotic response, but they are not only expressed as a result of fibrosis, they don't necessarily have common regulators and they can be expressed without expression of other ECM proteins or be derived from non-myofibroblast cells. For example, tumor cell-derived collagen III has been linked to a type of ECM that can maintain cancer dormancy (https://doi.org/10.1038/s43018-021-00291- 9). Here we provide a series of functional assays demonstrating that collagen VI can also be expressed and deposited by cancer cells and this is due to the absence of integrin-dependent mechanosensing and low levels of YAP signaling. While in primary tumours, that are also mainly stiff, collagen VI deposition is a part of a general desmoplastic response and could be deposited by stromal cells, cancer cells appear to express and secrete it in metastatic lesions where they confront a soft, naïve matrix. There is a reduction of metastasis by Collagen VI depletion in cancer cells. This was followed by reduced collagen VI staining in the formed metastatic lesions, suggesting that the main contributors to Collagen VI presence in metastasis are cancer cells. However, we also noticed that collagen VI was present to a degree in all metastatic lesions formed by Collagen VI KO cells, suggesting firstly that stromal cells can also deposit it there and secondly that metastasis can't be formed without collagen VI presence. We believe that our data and given the available tools, demonstrates both a mechanism for collagen VI expression and addresses its origin in the context of primary tumour and metastasis.

1. In terms of pathways sketched in Fig.2E and alluded to in the abstract, the idea that fibrillar collagen Col6 increases 'DUE to low YAP activity' is surprising and needs more evidence or qualification. I see in Fig.1D that the YAP target gene CTGF is low as expected on soft gels (but not 1C, curiously), but the effect on Col6 is puzzling. This is because the literature generally indicates CTGF is part of the fibrillar ECM and fibrosis pathway, and some studies indicates that includes COL6: "Collagen VI as a driver and disease biomarker in human fibrosis" [DOI: 10.1111/febs.16039]. Indeed, Fig.1D (and 1E) also shows higher fibronectin, which often precedes fibrillar collagen deposition.
Although soft matrix and siYAP show similar effects, both generally cause cell rounding (per Fig.S1A's 0.7kPa) and rounding can also trigger gene expression separate from direct regulation by YAP. At least as a thought experiment, if cells were confined on patterns that limited cell spreading, would Col6 be high and would it also increase/decrease with YAP knockdown/overexpression? What would happen to Fn1 and also Col1,3,5?
Put another way, do the authors believe YAP is not only a transcriptional (co)activator of CTGF but also a direct transcriptional (co)repressor of Fn1 and Col6 (and maybe Col1,3,5)? Could the latter be indirect effects? For example, the cells somehow sense a deficit in fibrillar ECM when they are rounded; alternatively, cell cycle is protracted on soft matrix (as is typical), and this leads to retention/accumulation of expressed genes that define lineage.

Our data doesn't imply that YAP is a direct repressor of Collagen VI, as we see all three Collagen VI genes increasing their expression upon loss of mechanosensing. However, this is not a generalised typical fibrotic response as other collagen genes do not change their expression in the same manner (Fig. S1F). Indeed, Ctgf, a known direct YAP target, is lower on soft matrix in both cell lines (See Fig. S1F, it is present on 1C also, but only certain gene names are shown for space reasons). This confirms the validity of the RNAseq and that gene expression in these cancer cells is tuned by mechanosensing. Cells on stiff polyacrylamide gels are not surrounded by fibrillar ECM. Fibronectin is covalently linked to the polyacrylamide hydrogels independent of stiffness, so cell rounding on soft ECM is not a result of the surrounding matrix but a direct response by lack of mechanosensing. Blockage of proliferation didn't increase (rather reduced) collagen VI expression in these

© 2022. Published by The Company of Biologists under the terms of the Creative Commons Attribution License (https://creativecommons.org/licenses/by/4.0/).

cells (Fig.S3B), so we don't have retention/accumulation but active increase of transcription and translation of collagen VI encoding genes.

2. What do Fig.4D,E look like for other fibrosis genes (CTGF, Col1,3,5, etc.)?

As our study is focussed on ColVI, we feel that investigating the prognostic potential of other pro-fibrotic genes is out of the scope of this study.

Second decision letter

MS ID#: JOCES/2022/259978

MS TITLE: Collagen-VI expression is negatively mechanosensitive in pancreatic cancer cells and supports the metastatic niche

AUTHORS: Vasileios Papalazarou, James Drew, Amelie Juin, Heather J Spence, Jamie Whitelaw, Colin Nixon, Manuel Salmeron-Sanchez, and Laura M. Machesky
ARTICLE TYPE: Research Article

We have now reached a decision on the above manuscript.

To see the reviewers' reports and a copy of this decision letter, please go to: https://submit-jcs.biologists.org and click on the 'Manuscripts with Decisions' queue in the Author Area. (Corresponding author only has access to reviews.)

As you will see, two reviewers are satisfied and one reviewer wants a specific single sentence amendment to the abstract. Please consider this request and explain your response in the cover letter; the point they are making seems relevant; the specific sentence may or may not be optimal. I hope that you will be able to address this single issue because I would like to be able to accept your paper. I will evaluate the edited abstract myself; it will not return to reviewers.

Please ensure that you clearly highlight all changes made in the revised manuscript. Please avoid using 'Tracked changes' in Word files as these are lost in PDF conversion.

I should be grateful if you would also provide a point-by-point response detailing how you have dealt with the points raised by the reviewers in the 'Response to Reviewers' box. Please attend to all of the reviewers' comments. If you do not agree with any of their criticisms or suggestions please explain clearly why this is so.

Reviewer 1

Advance summary and potential significance to field

Papalazarou and colleagues investigate how pancreatic tumor cells respond to their matrix environment. They define a mechanoresponsive biochemical pathway by which tumor cells upregulate expression of Collagen VI in the presence of a mechanically soft microenvironment. Using both in vitro and in vivo models the authors demonstrate that expression of ColVI by the tumor cells is required for invasion and metastasis, suggesting that the tumor cells may contribute to the increased desmoplasia that is a hallmark of PDAC and that likely contributes to its poor prognosis.

Comments for the author

The authors have thoroughly responded to the questions raised in the initial review. I have no additional concerns.

© 2022. Published by The Company of Biologists under the terms of the Creative Commons Attribution License (https://creativecommons.org/licenses/by/4.0/).

Reviewer 2

Advance summary and potential significance to field

The authors have done a great job in addressing all of my concerns. this is a really interesting study and is suitable to be published in JCS.

Comments for the author

none

Reviewer 3

Advance summary and potential significance to field

Per previous.

Comments for the author

The authors must write in their abstract and in their text exactly what they wrote in their response: "Our data doesn’t imply that YAP is a direct repressor of Collagen VI". This is essential because the abstract and Fig.2E continue to be deceptive on this point, and no data were added to clarify this important issue.

Second revision

Author response to reviewers' comments

We thank the reviewers for their positive comments and we have now made the recommended change from reviewer 3 in the abstract.

Third decision letter

MS ID#: JOCES/2022/259978

MS TITLE: Collagen-VI expression is negatively mechanosensitive in pancreatic cancer cells and supports the metastatic niche

AUTHORS: Vasileios Papalazarou, James Drew, Amelie Juin, Heather J Spence, Jamie Whitelaw, Colin Nixon, Manuel Salmeron-Sanchez, and Laura M. Machesky
ARTICLE TYPE: Research Article

I am happy to tell you that your manuscript has been accepted for publication in Journal of Cell Science, pending standard ethics checks. Your revised sentence was better than the reviewer's suggestion. I think their point was fair though. Thanks for getting it back quickly.

© 2022. Published by The Company of Biologists under the terms of the Creative Commons Attribution License (https://creativecommons.org/licenses/by/4.0/).
